# An Enhanced Prediction Model for Energy Consumption in Residential Houses: A Case Study in China

**DOI:** 10.3390/biomimetics10100684

**Published:** 2025-10-11

**Authors:** Haining Tian, Haji Endut Esmawee, Ramele Ramli Rohaslinda, Wenqiang Li, Congxiang Tian

**Affiliations:** 1 Research Center of Livable Construction of Human Settlements in Qinling-Daba Mountains, Shaanxi University of Technology, Hanzhong 723001, China; 2 Faculty of Built Environment, Universiti Teknologi MARA (UiTM), Shah Alam 40450, Selangor, Malaysia; 3 College of Built Environment, Universiti Teknologi MARA (UiTM), Perak Branch 32610, Perak, Malaysia

**Keywords:** improved BKA, CEC2017, residential buildings, energy consumption, prediction model, influencing factors

## Abstract

High energy consumption in Chinese rural residential buildings, caused by rudimentary construction methods and the poor thermal performance of building envelopes, poses a significant challenge to national sustainability and “dual carbon” goals. To address this, this study proposes a comprehensive modeling and analysis framework integrating an improved Bio-inspired Black-winged Kite Optimization Algorithm (IBKA) with Support Vector Regression (SVR). Firstly, to address the limitations of the original B-inspired BKA, such as premature convergence and low efficiency, the proposed IBKA incorporates diversification strategies, global information exchange, stochastic behavior selection, and an NGO-based random operator to enhance exploration and convergence. The improved algorithm is benchmarked against BKA and six other optimization methods. An orthogonal experimental design was employed to generate a dataset by systematically sampling combinations of influencing factors. Subsequently, the IBKA-SVR model was developed for energy consumption prediction and analysis. The model’s predictive accuracy and stability were validated by benchmarking it against six competing models, including GA-SVR, PSO-SVR, and the baseline SVR and so forth. Finally, to elucidate the model’s internal decision-making mechanism, the SHAP (SHapley Additive exPlanations) interpretability framework was employed to quantify the independent and interactive effects of each influencing factor on energy consumption. The results indicate that: (1) The IBKA demonstrates superior convergence accuracy and global search performance compared with BKA and other algorithms. (2) The proposed IBKA-SVR model exhibits exceptional predictive accuracy. Relative to the baseline SVR, the model reduces key error metrics by 37–40% and improves the R^2^ to 0.9792. Furthermore, in a comparative analysis against models tuned by other metaheuristic algorithms such as GA and PSO, the IBKA-SVR consistently maintained optimal performance. (3) The SHAP analysis reveals a clear hierarchy in the impact of the design features. The Insulation Thickness in Outer Wall and Insulation Thickness in Roof Covering are the dominant factors, followed by the Window-wall Ratios of various orientations and the Sun space Depth. Key features predominantly exhibit a negative impact, and a significant non-linear relationship exists between the dominant factors (e.g., insulation layers) and the predicted values. (4) Interaction analysis reveals a distinct hierarchy of interaction strengths among the building design variables. Strong synergistic effects are observed among the Sun space Depth, Insulation Thickness in Roof Covering, and the Window-wall Ratios in the East, West, and North. In contrast, the interaction effects between the Window-wall Ratio in the South and other variables are generally weak, indicating that its influence is approximately independent and linear. Therefore, the proposed bio-inspired framework, integrating the improved IBKA with SVR, effectively predicts and analyzes residential building energy consumption, thereby providing a robust decision-support tool for the data-driven optimization of building design and retrofitting strategies to advance energy efficiency and sustainability in rural housing.

## 1. Introduction

The world is facing issues such as energy crises, ecological destruction, and global warming. The construction industry accounts for approximately 40% of global energy consumption, making it a significant source of greenhouse gas emissions. Improving building energy efficiency benefits environmental protection and economic development [1]. With China’s economic growth and improved living standards, rural residents’ lifestyles, production methods, and behavioral patterns have undergone transformation. Modern household functions now prioritize comfort and amenities, and demands for improved residential environments and housing functionality are steadily increasing. However, current rural housing renovation and new construction practices generally prioritize aesthetics over functionality, leading to contradictions between architectural space design and residential sustainability. This approach fails to meet residents’ usage needs and increases dependence on air conditioning systems, significantly raising residential building energy consumption, which continues to grow as a proportion of total building energy use. This situation poses a significant challenge to the recommendations of the IPCC and China’s “dual carbon” strategic goals [2]. According to the Research Report on Carbon Emissions in Urban and Rural Construction in China (2024), in 2022, the total energy consumption of buildings and construction activities nationwide reached 2.42 billion tons of standard coal equivalent (tce), accounting for 44.8% of the country’s total energy consumption. The total carbon dioxide emissions from buildings and construction were 5.13 billion tons (tCO_2_), representing 48.3% of national energy-related carbon emissions. Specifically, rural residential building energy consumption was 250 million tce, or 10.33% of the national total, while the corresponding carbon emissions amounted to 480 million tCO_2_, or 9.36%. Therefore, conducting energy consumption predictions for residential buildings, thoroughly analyzing influencing factors, and implementing systematic optimization measures are not only crucial for promoting scientific rural housing updates and effectively controlling indoor energy consumption but also an inevitable requirement aligned with the concept of sustainable development. Scientific control of energy consumption in rural residential buildings can reduce energy waste and lower carbon emissions. While meeting current housing demands, it also ensures the long-term health of the ecological environment, achieving coordinated progress in economic development, improvement of people’s livelihoods, and environmental protection, thereby laying a solid foundation for the sustainable development of rural areas.

Therefore, developing a high-precision energy consumption prediction model is a foundational step toward addressing this challenge. Such a model serves as a powerful decision-support tool that can quantitatively simulate and compare the energy performance of various architectural schemes at the design stage. For existing buildings, it can diagnose sources of energy inefficiency and quantify the potential savings of different retrofitting measures, guiding homeowners toward the most cost-effective investments. Ultimately, this data-driven approach is essential for reducing energy waste and carbon emissions, thereby laying a solid foundation for sustainable development in rural areas.

Hence, a high-precision energy prediction model is foundational to this effort. It acts as a powerful decision-support instrument, turning abstract sustainability targets into concrete, operational plans. At the design stage, it empowers architects to simulate and contrast the energy profiles of various designs—for instance, with different insulation or window configurations—to lock in an optimal, low-energy solution from the start. For existing buildings, it helps diagnose inefficiencies and quantify the impact of potential upgrades, guiding owners toward cost-optimal retrofitting decisions. Fundamentally, the scientific oversight provided by these models drastically cuts energy waste and carbon emissions. This data-driven approach meets contemporary housing needs while ensuring long-term ecological integrity, fostering a coordinated advancement of economic, social, and environmental goals and thus creating a firm groundwork for sustainable development in rural regions. In recent years, scholars have employed surrogate models and their improved versions, such as extreme gradient boosting (XGBoost), random forest (RF), artificial neural network (ANN), gradient boosting decision tree (GBDT), and support vector regression (SVR), to establish prediction models [1,3,4,5]. Neo et al. proposed a hybrid XGBoost method that integrates building characteristics and urban landscape features to predict residential energy consumption in Singapore. They compared the method with a geographically weighted regression (GWR) model and a random forest (RF) model, verifying the effectiveness of the proposed approach [6]. L.G.B. Ruiz et al. presented a neural network-based prediction method to build an energy consumption prediction model for public buildings. They optimized the weights of the neural network using a genetic algorithm and then calculated the error values of the prediction model to verify its accuracy [7]. Chirag Deb et al. implemented linear regression and artificial neural network models to predict residential building energy consumption. By comparing the two models, they concluded that the neural network model provides more accurate predictions than the linear regression model [8]. Qi et al. combined the XGBoost, RF, and GBDT models and proposed an ensemble model suitable for household energy consumption prediction in high-density residential areas, and analyzed the key influencing factors based on this [9]. Guo et al. proposed a separated design method and process for energy-saving residential forms, and applied SVR to predict residential building energy consumption [10]. Zhussupbekov et al. analyzed the effectiveness of multiple regression (MR), support vector regression (SVR), and artificial neural networks (ANN) in predicting residential building energy consumption, demonstrating that the use of support vector machines for predicting residential building energy consumption is feasible [11].

Compared with other artificial intelligence technologies, support vector regression (SVR) prediction theory, which is based on small-sample statistical learning theory and the principle of structural risk minimization, demonstrates significant advantages in generalization capability, prediction accuracy, and model operational efficiency [12]. However, the primary limitations of SVR (Support Vector Regression) are twofold [13,14]: Firstly, SVR involves multiple sensitive parameters (e.g., the penalty coefficient C, kernel parameters) that necessitate manual adjustment. The absence of a standardized methodology for this tuning process means that suboptimal parameter selection can substantially degrade model accuracy. Secondly, SVR’s scalability is a concern, as its training on large-scale datasets results in a significant increase in both time complexity and memory usage, which in turn impairs training efficiency.

To improve the prediction accuracy of SVR, many scholars have applied various metaheuristic algorithms to determine the optimal values of SVR parameters [15]. Zhou et al. proposed a novel adaptive particle swarm optimization-support vector machine regression hybrid reliability prediction model, which effectively adjusts the parameters of the support vector machine regression and enhances its accuracy [15]. Yong et al. investigated the optimization effects of particle swarm optimization, whale optimization algorithm, differential evolution, and covariance matrix adaptation evolution strategy on SVR. The results indicated that the SVR-PSO model achieved the highest accuracy, followed closely by the SVR-DE and SVR-CMAES models [16]. Xu et al. introduced a new ensemble modeling method for predicting peak particle velocity (PPV) and frequency based on multivariate adaptive regression splines (MARS), support vector regression (SVR), and the black widow optimization algorithm (BWOA). The results showed that the model optimized by the black widow optimization algorithm outperformed the unoptimized version [17]. Li et al. proposed an innovative algorithm combining the Harris hawk optimization (HHO) algorithm and support vector regression (SVR) for dam health monitoring modeling [18]. Li et al. proposed an improved sine cosine algorithm (SCA) incorporating strategies such as good point set initialization of the population, nonlinear amplitude adjustment factor, random inertia weight, and adaptive endpoint weights. This modified SCA was used to optimize the support vector regression (SVR) model. Experimental results indicate that the optimized target threat estimation model demonstrates high accuracy and stability [19]. Li et al. employed support vector machines to establish a prediction model for the performance of hardened concrete, and optimized the parameters of the SVR using the marine predators algorithm (MPA) and grasshopper optimization algorithm (GOA). The results showed that the MPA–SVR model outperforms the GOA–SVR [20]. Lynda et al., by integrating the dragonfly algorithm with SVR, proposed a method for modeling the nonlinear behavior in thin-layer drying kinetics of pea pods. Their results confirm that the DA–SVM technique can reliably describe the thin-layer drying kinetics of pea pods [21]. Seyed Babak et al. compared the performance of several algorithms—support vector regression (SVR), support vector regression–firefly algorithm (SVR–FA), support vector regression–grasshopper optimization algorithm (SVR–GOA), and support vector regression–artificial bee colony (SVR–ABC)—in predicting E20 in stepped cascades [22]. Wang et al. developed a compressive strength prediction model for ultra-high-performance concrete (UHPC) based on SVR, and further optimized the support vector regression (SVR) model using the arithmetic optimization algorithm (AOA), The superior performance and computational efficiency of the AOA-SVR model in predicting the compressive strength of UHPC were verified. Results indicate that the optimized AOA-SVR model exhibits strong generalization capability and can more accurately predict the compressive strength of UHPC [23]. Hameed et al. proposed a hybrid framework combining an improved grey wolf optimizer (IGWO) with support vector regression (SVR) for wind resource estimation and forecasting. Results demonstrate that the proposed model provides the best evaluation report, with assessment metrics indicating its superior accuracy in predicting wind behavior [24]. Hou et al. constructed a sparrow search algorithm-incremental support vector regression (SSA-ISVR) model for online concrete temperature prediction. The penalty coefficient and kernel coefficient of the ISVR algorithm were optimized using SSA. Results show that the SSA-ISVR model outperforms LSTM, BP, and ISVR models [25].

In essence, metaheuristics are the dominant method for tuning SVR parameters. However, classic algorithms like PSO and GA face saturated research and diminishing returns on structural innovation. Critically, their performance is hampered by static control parameters that demand laborious, problem-specific adjustments, undermining their adaptability. This has spurred the exploration of novel bio-inspired algorithms. The Black Kite Algorithm (BKA), recently proposed by Wang and its team [26] based on the intelligent behaviors of its natural counterpart, presents a significant advancement. It demonstrates superior optimization, convergence, and performance over many existing methods. BKA’s strength lies in its intrinsic mechanism for balancing local exploitation and global exploration, bypassing the need for static parameter tuning. As an emerging algorithm, BKA is an active area of current research. Thus, selecting BKA for targeted improvement is not only a novel research direction but also a valuable endeavor to broaden its practical utility. The main contributions of this study are as follows:

A framework for modeling and analyzing residential building energy consumption is introduced, combining an enhanced Bio-inspired Black-winged Kite Optimization Algorithm (IBKA) with Support Vector Regression (SVR). A multi-strategy approach is adopted to enhance the performance of the original Bio-inspired BKA, and the improved algorithm is utilized to obtain the optimal parameters for SVR. The newly proposed IBKA-SVR model is applied to predict residential building energy consumption, demonstrating higher prediction accuracy compared to traditional SVR. The main contributions of this study are as follows:

(1) An improved Black-winged Kite Optimization Algorithm is proposed, which enhances the optimization efficiency and global search capability of the original algorithm. The optimization performance of the improved algorithm is verified using the CEC2017 benchmark test functions;

(2) A regression modeling method based on IBKA-SVR is introduced, where the IBKA algorithm optimizes the parameters of SVR, thereby improving the accuracy of regression modeling;

(3) The IBKA-SVR regression model is employed to establish a residential building energy consumption prediction model, the SHAP (SHapley Additive exPlanations) interpretability framework was employed to quantitatively elucidate the impact of both the independent contributions and the interaction effects of each influencing factor on energy consumption.

The structure of the paper is as follows: In Section 2, the authors briefly introduce the fundamental principles of the Black-winged Kite Optimization Algorithm, the improvement strategies, and the performance of the improved algorithm on the CEC2017 benchmark test functions. Section 3 provides a detailed description of the basic principles of SVR and the design framework of the IBKA-SVR-based regression model. In Section 4, a residential building energy consumption simulation model is established. The IBKA-SVR regression model is used to build the residential building energy consumption prediction model, and using the SHAP analysis framework, we quantitatively clarified the independent and interactive effects of each influencing factor on energy consumption. Section 5 presents a thorough analysis and discussion of the research findings. Section 6 summarizes the main conclusions of this study and provides relevant recommendations based on the limitations of the current research and future directions.

## 2. Improved Black-Winged Kite Optimization Algorithm

### 2.1. Black-Winged Kite Optimization Algorithm

(1) Initialization Phase

In the BKA algorithm, during the initialization phase, each black-winged kite’s position (representing a potential solution) is determined as follows. The population positions are represented using a matrix:

(1) $$BK=\left[\begin{array}{l} BK_{1,1}\;BK_{1,2}\;\ldots \;\ldots \;BK_{1,dim}\\ BK_{2,1}\;BK_{2,2}\;\ldots \;\ldots \;BK_{2,dim}\\ \;\vdots \;\;\;\vdots \;\vdots \;\vdots \;\;\vdots \\ \;\vdots \;\;\;\vdots \;\vdots \;\vdots \;\;\vdots \\ BK_{pop,1},BK_{pop,2}\;\ldots \;\ldots \;BK_{pop,1} \end{array}\right]$$

Among them, pop represents the population size (the number of potential solutions); dim denotes the problem dimension, and $BK_{i,j}$ indicates the position of the i-th Black-winged Kite in the j-th dimension.

The position of each Black-winged Kite is uniformly distributed within the given range. The calculation formula is:

(2) $$X_{i}=BK_{lb}+rand\left(BK_{ub}-BK_{lb}\right)$$

Here, $BK_{lb}$ and $BK_{ub}$ represent the lower and upper bounds of the i-th Black-winged Kite in the j-th dimension, respectively, and rand is a randomly selected value within the interval [0, 1].

Initial leader selection: During initialization, the individual with the highest fitness value in the population is selected as the leader. Taking minimization as an example, the mathematical expression is:

(3) $$f_{best}=\min\left(f\left(X_{i}\right)\right)$$

(4) $$X_{L}=X\left(find\left(f_{best}==f\left(X_{i}\right)\right)\right)$$

$f_{best}$ is the minimum fitness value of individuals in the population, and $X_{L}$ is the position of the individual corresponding to the optimal fitness value, i.e., the initial leader’s position.

(2) Attacking Behavior

Black-winged kites adjust their attacking strategies according to different situations during predation. Reflected in the algorithm, their position update formula is as follows:

(5) $$y_{t+1}^{i,j}=\left\{\begin{array}{l} y_{t}^{i,j}+n\times \left(1+\sin\left(r\right)\right)\times y_{t}^{i,j}\;p<r\\ y_{t}^{i,j}+n\times \left(2r-1\right)\times y_{t}^{i,j}\;\;\;\;else \end{array}\right.$$

Here, $y_{t}^{i,j}$ and $y_{t+1}^{i,j}$ denote the positions of the i-th black-winged kite in the j-th dimension at time t and t+1, respectively; r is a random number between 0 and 1; p is a constant with a value of 0.9; T represents the total number of iterations, and t is the current number of completed iterations.

(3) Migration Behavior

Since bird migration is influenced by multiple factors and is typically led by leaders, the mathematical model of the migration behavior of Black-winged Kite (BKA) in the algorithm is as follows:

(6) $$y_{t+1}^{i,j}=\left\{\begin{array}{l} y_{t}^{i,j}+C\times \left(0,1\right)\times \left(y_{t}^{i,j}-L_{t}^{j}\right)\;\;F_{i}<\;F_{ri}\\ y_{t}^{i,j}+C\times \left(0,1\right)\times \left(L_{t}^{j}-m\times y_{t}^{i,j}\right)\;else \end{array}\right.$$

(7) $$m=2\times \sin\left(r+\pi /2\right)$$

Here, $L_{t}^{j}$ denotes the leading score of the Black-winged Kite in the j-th dimension at the t-th iteration; $F_{i}$ is the fitness value of the i-th Black-winged Kite in the j-th dimension at the t-th iteration; $F_{ri}$ is the fitness value obtained from a random position in the j-th dimension by any Black-winged Kite at the t-th iteration; and C(0,1) represents the Cauchy mutation. The one-dimensional probability density function of the Cauchy distribution for the Cauchy mutation is given as:

(8) $$f\left(x,\delta ,\mu \right)=\frac{1}{\pi }\times \frac{\delta }{\delta^{2}+{\left(x-\mu \right)}^{2}},\;-\infty <x<\infty$$

(9) $$f\left(x,\delta ,\mu \right)=\frac{1}{\pi }\times \frac{1}{x^{2}+1},\;-\infty <x<\infty$$

### 2.2. Multi-Strategy Improved BKA Algorithm

- *Improvement 1: A random selection mechanism for attack behaviors.*

The original BKA determines attack patterns using a fixed threshold (*p* = 0.9), which induces behavioral bias and imbalanced exploration; regions associated with low-probability behaviors are systematically neglected, making the algorithm prone to premature convergence and to missing global optima on complex problems. To address this, we reconstruct the decision model based on uniformly distributed random numbers rand~U(0,1) and adopt an equal-probability (50%) dual-behavior selection strategy, as presented in Equation (10), thereby achieving a dynamic balance between exploration and exploitation that enhances early-stage global coverage and late-stage local optimization efficiency. This strategy is problem-agnostic; through a generic probability-balancing mechanism, it improves the algorithm’s generality, randomness, and solution performance.

(10) rand<0.5

- *Improvement 2: Position Update Mechanism Integrated with Global Information*

Existing BKA relies solely on information from each individual and the leader, disregarding population-level distributional characteristics; as a result, it is prone to unguided search, premature convergence, and inefficient iterations. To address this, we incorporate a global information model into the position-update mechanism. This model is built upon two key population statistics: the population mean (μ), calculated as shown in Equation (11), and the median (median(X)), defined in Equation (12). The mean steers solutions toward the population centroid, suppressing divergence and improving search directionality and rate, whereas the median provides a robust estimate of central tendency in the presence of outliers. By working synergistically, these two statistics, as defined in Equations (11) and (12), expose individuals to global structural cues, yield a better exploration–exploitation balance, strengthen the ability to escape local optima, and markedly improve the convergence efficiency, stability, and overall performance of BKA.

(11) $$\mu =\frac{1}{N}\sum_{i=1}^{N}X_{i}^{j}$$

(12) $$median(X)=median(X_{1}^{j},X_{2}^{j},\ldots ,X_{N}^{j}),\;j=1,\ldots ,d$$

- *Improvement 3: A mechanism for escaping local optima based on random neighborhood perturbation.*

To address the tendency of the original BKA algorithm to become trapped in local optima, we introduce a random learning mechanism. This mechanism involves updating the current individual’s position using information from another individual, $x_{t}^{k,j}$, randomly selected from the population. The index k of the selected individual is generated according to Equation (13). This random learning strategy is then integrated into the comprehensive position update formula for the attack phase, as detailed in the second condition of Equation (14). By incorporating this component, the algorithm injects randomness that enables individuals to move in diverse directions, increasing the likelihood of escaping local traps. Furthermore, it promotes information sharing and diffusion within the population, as individuals can leverage information from peers, not just the leader or population statistics. This allows the algorithm to more fully exploit collective intelligence, explore unknown regions, and increase the probability of discovering superior solutions.

(13) k=randi(1,N)

The improved BKA algorithm, based on comprehensive improvements 1–3, updates the position during the attack phase according to the following formula:

(14) $$y_{t+1}^{i,j}=\left\{\begin{array}{l} \;y_{t}^{i,j}+D\times \left[r\times (X_{mean}-y_{t}^{i,j})+r\times (X_{mean}-X_{median}^{j})\right]\;\;rand<0.5\\ y_{t}^{i,j}-x_{t}^{k,j}+L_{t}^{j}\;\;\;\;\;\;\;\;\;\;\;\;\;\;\;\;\;\;else \end{array}\right.$$

(15) $$D=2-e^{-\frac{t^{2}}{T\times rand}}$$

Among them, rand is a uniform random number within the interval [0, 1].

- *Improvement 4: Integration of the NGO random selection operator.*

To enhance search diversity and mitigate homogeneous migration, we draw inspiration from the exploration stage of Northern Goshawk Optimization (NGO). We introduce its random selection operator, denoted as I, which is designed to inject randomness into individual migration displacements. This operator is formally defined in Equation (16), where it randomly takes a value of either 1 or 2.

(16) I=round(1+rand)

Subsequently, this operator is incorporated into the position update formula for the migration phase of the BKA, as detailed in Equation (17).

(17) $$y_{t+1}^{i,j}=\left\{\begin{array}{ll} x_{t}^{i,j}+C\times \left(x_{t}^{i,j}-I\times L_{t}^{j}\right) & F_{i}<F_{ri}\\ x_{t}^{i,j}+C\times \left(L_{t}^{j}-m\times x_{t}^{i,j}\right) & else \end{array}\right.$$

Here, $C=\;\mathrm{clip}\;(\tan((U-\frac{1}{2})\pi ),-10,10)$.

As shown in the first condition of Equation (17), the value of I directly alters the step size calculation, producing heterogeneous steps and directions. This mechanism promotes population diversity in the search space, improves the trade-off between global exploration and local exploitation, and enhances the overall global optimization performance.

**Figure 1:**
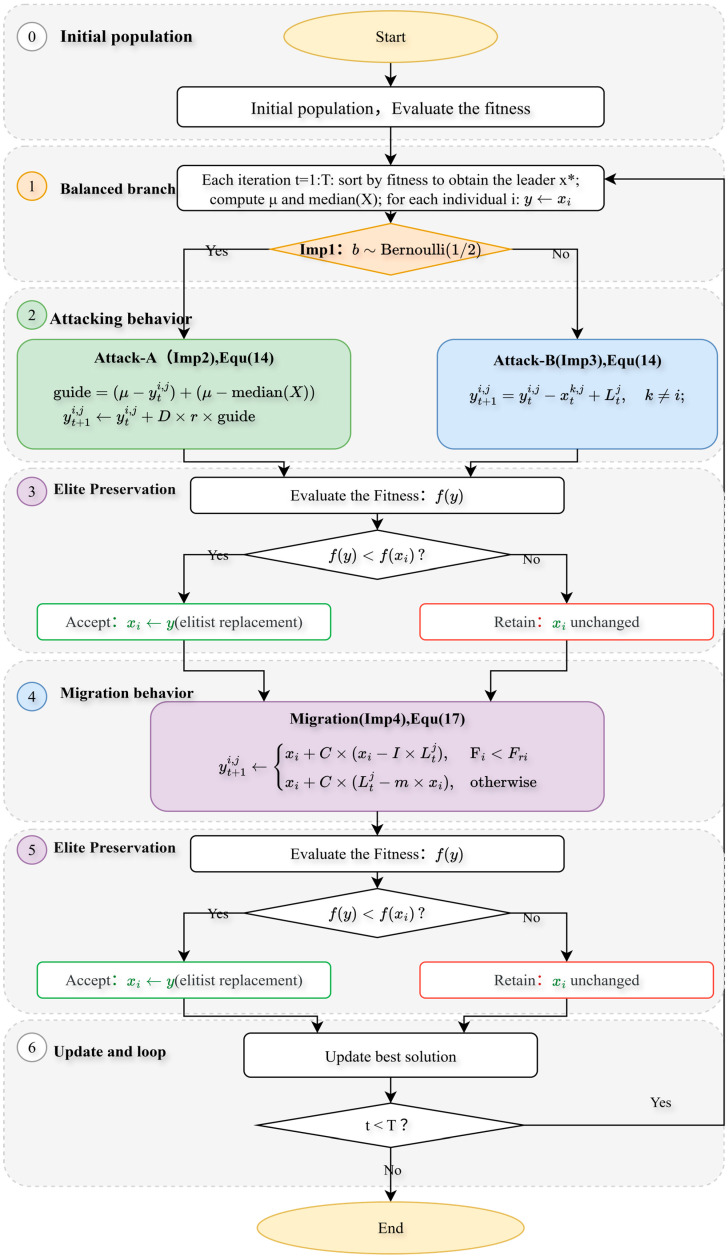
Flowchart of the improved BKA algorithm.

Flowchart of the improved BKA algorithm is shown in Figure 1.

### 2.3. Stability Analysis of the IBKA Algorithm

**Theorem 1.**  *Let the objective function *f:Ω→ℝ *be measurable on a compact and convex set* $\Omega \subset {\mathbb{R}}^{d}$ *, and let it be lower-bounded by* $f^{*}$ *. Suppose the EBKA algorithm is improved with four strategies, and at each iteration, candidate points are projected or clipped back onto the set* Ω *. Then the sequence of historical bests,* $\left\{x_{\mathrm{best}}^{t}\right\}$*, satisfies the following:*

(1) $f_{\mathrm{best}}^{t}:=f(x_{\mathrm{best}}^{t})$* is monotonically non-increasing and bounded below, and therefore converges almost surely (a.s.) to a finite limit *$f_{\infty }\geq f^{*}$.
(2) *The second moment of the single-step displacement *$E[\|x_{t+1}-x_{t}{\|}^{2}|F_{t}]\leq M^{2}$* is bounded: *M<∞ *is a constant.*

**Proof.** 

(1) Monotonicity and Boundedness

All four improvements are implemented with an elitist selection criterion: a solution is replaced only if a new solution is superior. Therefore,

(18) $$f_{\mathrm{best}}^{t+1}\leq f_{\mathrm{best}}^{t}\geq f^{*}$$

The sequence $\left\{f_{\mathrm{best}}^{t}\right\}$ is monotonically non-increasing. Since it is also bounded below, the limit $f_{\infty }$ is guaranteed to exist.

(2) Bounded Second Moment of One-Step Displacement

Let $D:=\underset{x,y\in \Omega }{\sup}\|x-y{\|}_{2}$ be the diameter of the domain, and let $R:=\underset{z\in \Omega }{\sup}\|z{\|}_{2}$ (which is finite).

- Attack Step (Executed with 50% probability after an equal-probability selection, this step performs global guidance)

Let μ be the component-wise mean, m be the component-wise median, and $\xi \in {\left[0,1\right]}^{d}$ be a component-wise independent scaling factor. C∈(1,2). Then

(19) Δx=C⋅ξ⋅[(μ−x)+(m−x)], ξ~U(0,1)

Due to $\mu =\frac{1}{N}\sum x_{i},\;m=\mathrm{median}(x_{i}),x\in \Omega$, and ${\left\|\mu -x\right\|}_{2}\leq D,\;{\left\|\mu -m\right\|}_{2}\leq D$ then

(20) $${\left\|\Delta x\right\|}_{2}\leq 2(D+D)\leq 4D$$

- Neighborhood Perturbation Step (Executed with the other 50% probability)

(21) $$\Delta x=-x_{s}+x_{\mathrm{leader}},x_{s},x_{\mathrm{leader}}\in \Omega \Rightarrow {\left\|\Delta x\right\|}_{2}\leq D$$

- Migration Step (Based on NGO + truncated Cauchy distribution)

Let $s={\mathrm{clip}}_{K}[\tan(\pi (u-1/2))]$, |s|≤K, and the direction be taken as:

(22) $$h(x)\in \{x-I\cdot x_{\mathrm{best}},\;x_{\mathrm{best}}-m\cdot x\},I\in \{1,2\},\;|m|\leq 2$$

and $\|h(x){\|}_{2}\leq (1+I)R\leq 3R$, then

(23) $$\|\Delta x{\|}_{2}=|s|\cdot \|h(x){\|}_{2}\leq 3KR$$

Due to M:=max{4D,D,3KR}, then

(24) $$E[\|x_{t+1}-x_{t}{\|}_{2}^{2}|F_{t}]\leq M^{2}$$

(3) Non-Starvation and Opportunity

The 50/50 branch selection is an independent Bernoulli (1/2) trial, which, per the Borel–Cantelli lemma, ensures both attack branches are executed infinitely often almost surely; the migration step is also executed each round. This guarantees infinite attempts for each operator, though not that a single step can cover the entire search space Ω. □

In conclusion, EBKA remains monotonically bounded with a bounded second moment despite its four-heuristic structure. It thus fulfills the conditions of the fundamental convergence framework, ensuring stability and convergence.

### 2.4. Ablation Study on the Four Improvements (CEC2017, D = 30)

**Figure 2:**
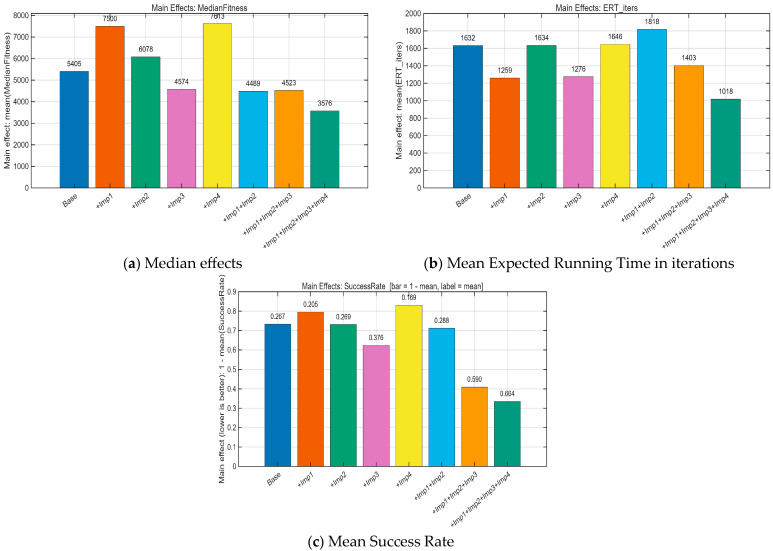
Main Effects Analysis.

**Figure 3:**
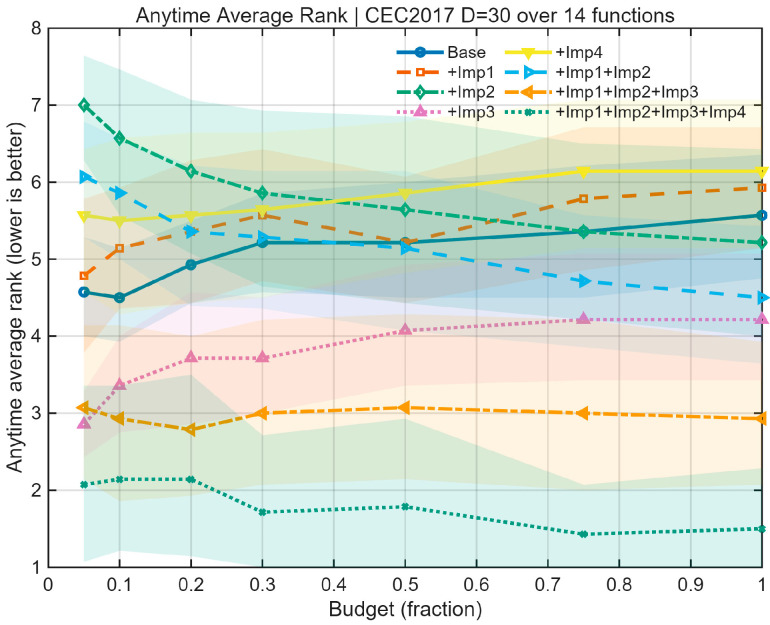
Anytime Average Rank.

**Figure 4:**
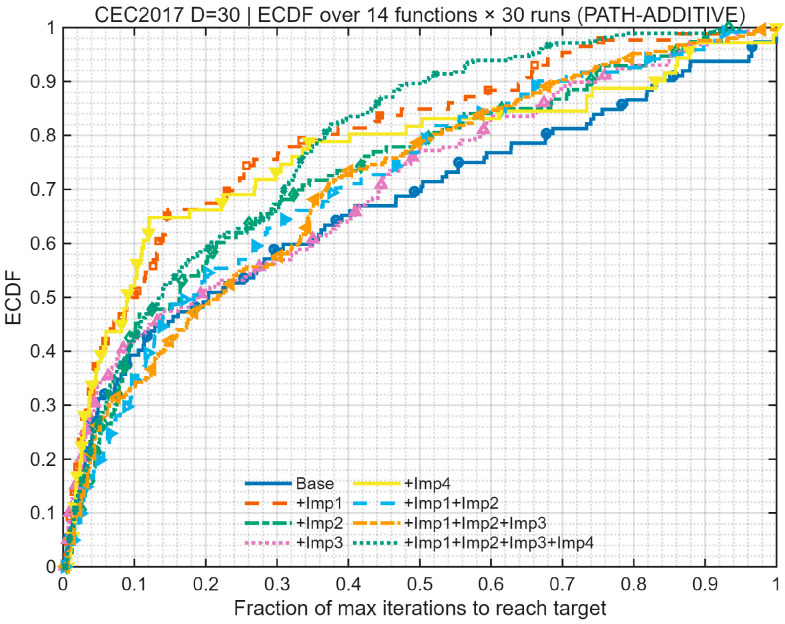
Time-To-Target (ECDF).

We performed an ablation study employing a “path-additive” methodology to assess eight algorithmic variants: Base, +Imp1, +Imp2, +Imp3, +Imp4, +Imp1+Imp2, +Imp1+Imp2+Imp3, and Full (+Imp1+Imp2+Imp3+Imp4). The experiments were conducted under uniform settings: population = 30, dimensions D = 30, and a maximum budget of 10,000 × D function evaluations (MaxFEs), with 2 stages per generation. A selection of CEC 2017 benchmark functions (F1, F3, F4, F6, F8, F11, F13, F15, F18, F21, F23, F26, F28, F30) was used for testing, with 30 independent runs per function under fixed random seeds. Performance was evaluated based on Average Rank, Number of Wins/Ties/Losses, Mean Success Rate (Ps), Mean Expected Running Time in iterations (ERTi_mean) and evaluations (ERTe_mean), Time-To-Target (ECDF), Anytime Average Rank (AAR), and Main Effects Analysis. The outcomes of this ablation experiment are illustrated in Figure 2, Figure 3 and Figure 4 and Table 1.

The ablation study results in Figure 2, Figure 3 and Figure 4 and Table 1 show that the baseline BKA has moderate performance. Introducing Imp1 alone improves speed but compromises stability and final accuracy. Imp2 alone is largely detrimental, increasing instability. In contrast, Imp3 is a critical component, simultaneously enhancing robustness, efficiency, and solution quality. Imp4 by itself suppresses performance. When combined, Imp1+Imp2 improves success rate but slows the algorithm. The addition of Imp3 (+Imp1+Imp2+Imp3) creates a strong balance of speed and stability. Finally, the full model incorporating all four improvements (Imp1+Imp2+Imp3+Imp4) demonstrates clear superiority, achieving the best results across all metrics (Ps +39.7%, ERT −37.6%, Median −33.9%) and winning on every benchmark function. This synergy is further confirmed by the Anytime Average Rank and ECDF plots, which show the full model’s consistent dominance.

### 2.5. Improved Performance of the Black-Winged Kite Optimization Algorithm

**Figure 5:**
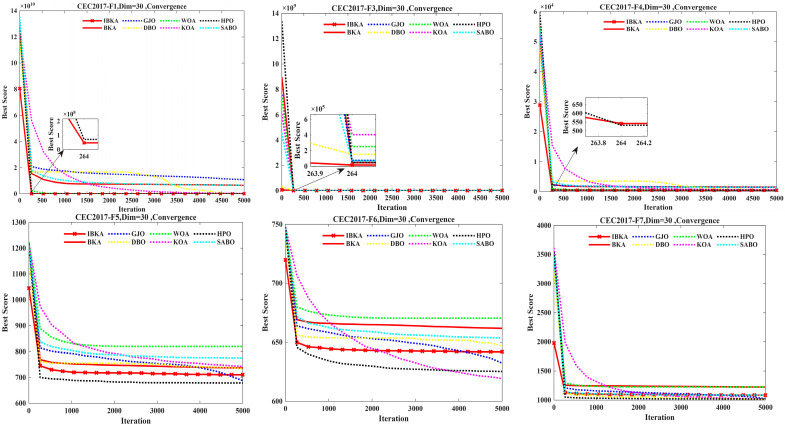

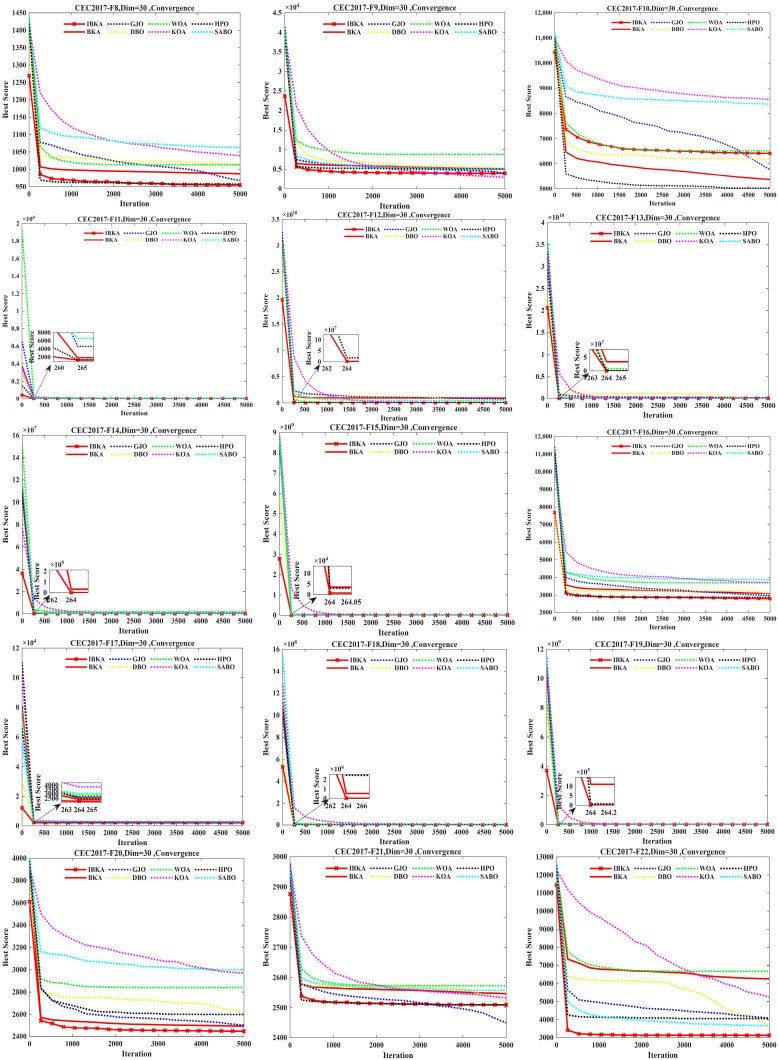

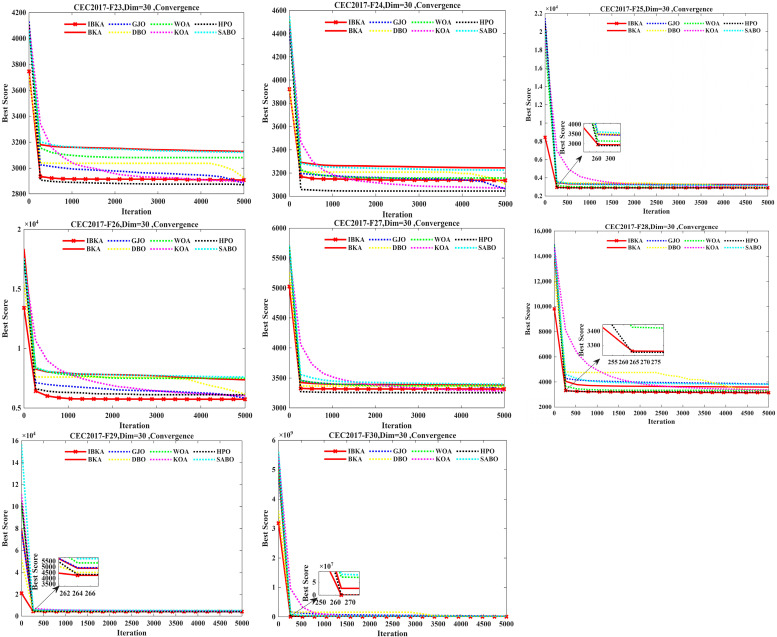
Convergence Curves of 8 Algorithms.

**Figure 6:**
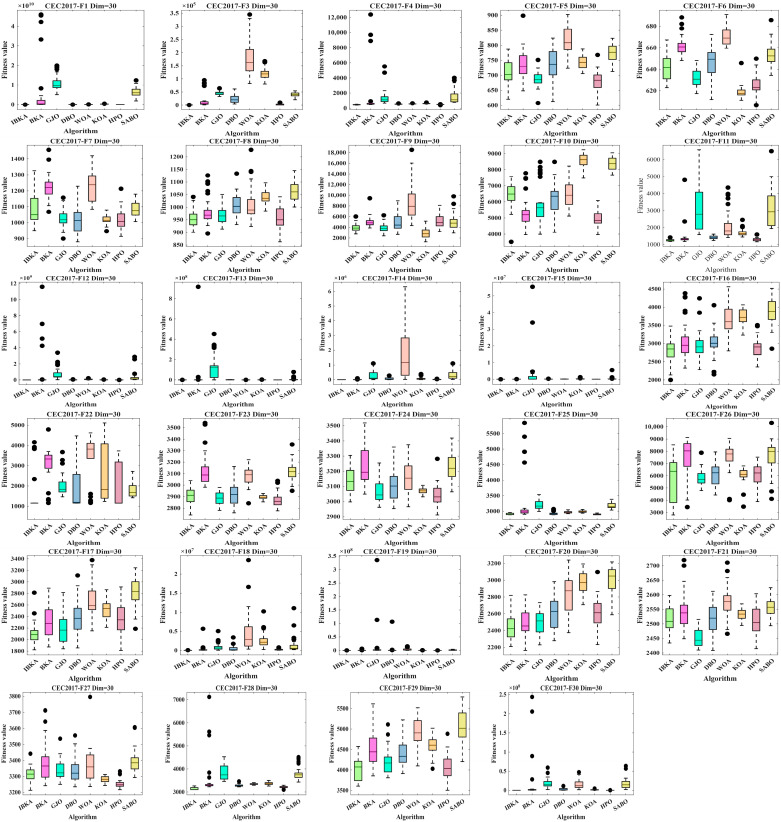
Boxplots of 8 Algorithms.

Comparison Algorithms and Parameter Settings. To verify the performance of the IBKA algorithm, this paper selects the BKA, GJO, DBO, WOA, KOA, HPO, and SABO algorithms for comparative experiments. The test functions are the 29 standard functions from CEC2017. To ensure fairness in the experiments, each of these eight algorithms is executed 30 times independently on every test function. The population size (pop) is set to 30, the maximum number of iterations (Tmax) is set to 5000, and the algorithm performance is evaluated under 30-dimensional conditions. The mean (avg), standard deviation (std), median, Wilcoxon test *p*-values, and the results of the Wilcoxon Rank-Sum Test are presented in Table 2. The convergence curves are shown in Figure 5, and the box plots are depicted in Figure 6.

The results in Table 2 indicate that the Friedman rank-sum test places the IBKA algorithm first among the eight algorithms with an average rank of 1.97. The complete ranking is as follows: IBKA (1) > HPO (2) > DBO (3) > GJO (4) > KOA (5) > BKA (6) > WOA (7) > SABO (8). This demonstrates that IBKA holds a significant overall advantage in optimization performance, showcasing superior adaptability and stability.

On test functions F1, F3, F12, F13, F14, and F15, IBKA significantly outperforms other algorithms across all three metrics: mean (avg), standard deviation (std), and median. Its mean and standard deviation are often several orders of magnitude lower than those of the competing algorithms. For instance, on F1, IBKA’s mean (1.04 × 10^2^) is two orders of magnitude better than the second-best algorithm, HPO (8.60 × 10^3^), and seven orders of magnitude better than the original BKA (6.49 × 10^9^). The Wilcoxon test *p*-values, consistently at 3.02 × 10^−11^ or a similar magnitude, confirm that IBKA’s performance advantage is extremely statistically significant. This suggests that when tackling high-dimensional, complex landscapes, IBKA’s global search capability and convergence speed are qualitatively enhanced, enabling it to effectively avoid premature convergence and consistently locate regions near the global optimum.

On test functions F4, F5, F6, F7, F8, F9, F10, and F11, IBKA ranks highly in terms of overall performance. Although it may not be first on every single metric for some functions, its overall superiority is evident. On F4, F8, F9, and F11, IBKA’s mean ranks in the top two, showcasing its high precision. On F4, F5, and F11, its standard deviation is excellent, indicating highly stable algorithm performance. With few exceptions (e.g., *p* = 0.0679 against HPO on F4; *p* = 0.483 against SABO on F7), IBKA shows a statistically significant difference (*p* < 0.05) compared to most algorithms, demonstrating the strong generalizability of the proposed strategies. The lack of significant difference in a few isolated cases is consistent with the “No Free Lunch” theorem and does not detract from its overall dominance. Across these 15 high-dimensional complex functions, IBKA demonstrates a significant or absolute advantage on 14 of them. Particularly on functions like F1, F3, and F12-F15, IBKA shows revolutionary performance improvements, providing powerful evidence that the proposed strategies fundamentally enhance BKA’s ability to solve complex optimization problems.

On test functions F17, F18, F19, F20, F22, F28, F29, and F30, IBKA’s mean rank is within the top three, with a particularly pronounced advantage on F17, F18, F19, F28, and F30. For example, on F30, IBKA’s mean (1.07 × 10^4^) is nearly 40% better than the second-best performer, HPO (1.78 × 10^4^). On F18, F19, F28, and F30, the *p*-values against all competitors are less than 0.05, indicating a significant advantage. While not statistically different from a few algorithms on F17, F20, F22, and F29, its metric values are more competitive. This shows that IBKA not only excels at global exploration but also possesses outstanding local exploitation capabilities and the ability to escape local optima, allowing it to find high-quality solutions in complex multi-modal environments.

On test functions F16, F21, F23, F24, F25, F26, and F27, IBKA’s mean rank is typically third or fourth, indicating moderate performance. However, its standard deviation is generally better, signifying high robustness and low performance fluctuation. On several functions, IBKA shows no significant difference from multiple algorithms (e.g., BKA, GJO, HPO on F16; GJO, DBO, KOA, HPO on F26), forming a group of statistically comparable algorithms. While IBKA does not achieve a significant win here, it also does not lag behind. Across this function set, IBKA leads or is highly competitive on more than half (8) of them, while maintaining stable performance on the rest. This indicates that IBKA is a well-balanced algorithm with no apparent weaknesses.

In a direct comparison between IBKA and BKA, IBKA outperforms BKA on the mean value for 28 functions, with precision improvements of several orders of magnitude on 20 of them (e.g., a 7-order-of-magnitude improvement on F1). It achieves a lower standard deviation on 26 functions, indicating significantly enhanced stability. The Wilcoxon rank-sum test shows that IBKA is significantly better than BKA on 27 functions (93.1%) at a *p* < 0.05 significance level. Ultimately, IBKA’s Friedman average rank (1.97) is far superior to BKA’s (6.21), statistically validating the effectiveness of the proposed improvement strategies.

As illustrated in Figure 5, IBKA demonstrates a significant comprehensive advantage, which is primarily attributed to its enhanced and well-balanced capabilities in both exploration and exploitation. This synergistic advantage directly translates into its exceptional performance in optimization accuracy. Out of the 29 test functions in the CEC2017 suite, IBKA achieved optimal or near-optimal solution accuracy on 18 functions (F1, F3, F8, F11–F20, F22, F26, F28–F30). In contrast, on functions F4, F5, F6, F7, F9, F10, F21, F23, F24, F25, and F27, its accuracy was slightly inferior to algorithms like HPO and GJO, which exhibit more aggressive exploitation phases. Crucially, the improvement of IBKA over the original BKA is fundamental. On 28 of the 29 CEC2017 functions, IBKA’s accuracy was superior to or on par with that of BKA, underperforming only in the isolated case of F10. This indicates that the proposed strategies not only enhance exploration to successfully overcome the “premature convergence” issue in BKA—caused by an early loss of population diversity—but also strengthen its capacity for sustained exploitation in high-quality solution regions. Consequently, IBKA demonstrates excellent rapid convergence, enabling it to locate the global optimum both quickly and accurately. Overall, the improved IBKA algorithm not only represents a substantial enhancement over the original BKA but also exhibits stronger comprehensive competitiveness than other state-of-the-art algorithms in terms of convergence accuracy, rapid convergence, and the exploration-exploitation balance.

As shown in Figure 6, across the 29 test functions in the CEC2017 suite, the IBKA algorithm consistently maintained a relatively small median on functions F1–F4, F11, F12–F20, F22, F25, F27, F28, and F30. On the remaining functions, its performance was second to that of algorithms such as KOA, HPO, and GJO. When compared to BKA, the IBKA algorithm achieved a smaller median on all functions with the sole exception of F10. Therefore, this demonstrates that the improvement strategies proposed in this paper result in a significant enhancement in optimization performance over the original BKA algorithm.

## 3. Prediction Model for Energy Consumption

### 3.1. Model Selection

**Figure 7:**
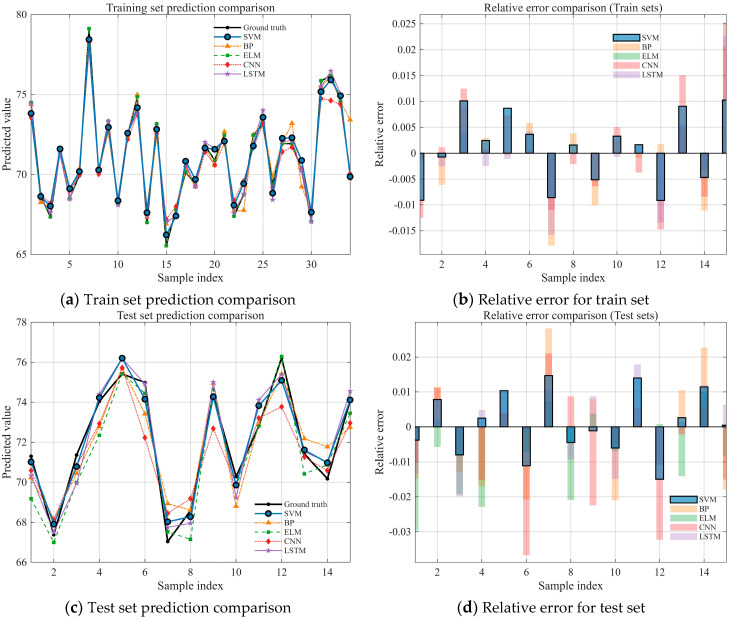

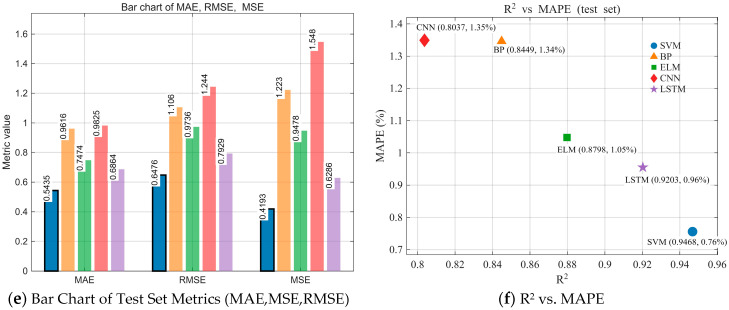
Evaluation Index of Seven basic Models for Energy Consumption Prediction.

To evaluate the suitability of different models for the research subject of this study, we conducted a fundamental comparison of five methods: Support Vector Regression (SVR, denoted as SVM in the tables), Backpropagation Neural Network (BP), Extreme Learning Machine (ELM), Convolutional Neural Network (CNN), and Long Short-Term Memory network (LSTM). The comparison was performed under a unified framework encompassing the same dataset, feature processing, and evaluation pipeline. Performance was assessed using six metrics: MAE, MAPE, MSE, RMSE, R^2^, and Runtime. The fitting results and evaluation metrics are presented in Figure 7 and Table 3.

As indicated by Figure 7 and Table 3, SVR demonstrates the best overall performance across all six metrics: MAE = 0.5435, MAPE = 0.7561, MSE = 0.4193, RMSE = 0.6476, and R^2^ = 0.9468, while also having the lowest computational cost (Runtime = 0.0056 s). Compared to the runner-up, LSTM, SVR reduces MAE and RMSE by approximately 20.8% and 18.3%, respectively, decreases MSE by about 33.3%, and achieves an absolute improvement in R^2^ of 0.0265 (a relative increase of ~2.9%), all while being approximately 960 times faster in inference speed. In comparison to ELM, SVR significantly improves accuracy (an absolute R^2^ increase of 0.067 and a ~33.5% decrease in RMSE) while maintaining a similar order of magnitude in computational cost and being ~36% faster. Relative to BP and CNN, SVR decreases RMSE by approximately 41.4% and 47.9% and increases R^2^ by 0.102 and 0.143, respectively, while simultaneously reducing runtime by more than two orders of magnitude. These results indicate that, for the current sample size and noise level, SVR effectively captures the non-linear relationships using kernel methods. Furthermore, by leveraging structural risk minimization and regularization, it achieves superior generalization and stability. Consequently, SVR significantly outperforms the other candidate models in both accuracy and efficiency and was therefore selected as the predictive modeling method.

### 3.2. Basic Principles of SVR

**Figure 8:**
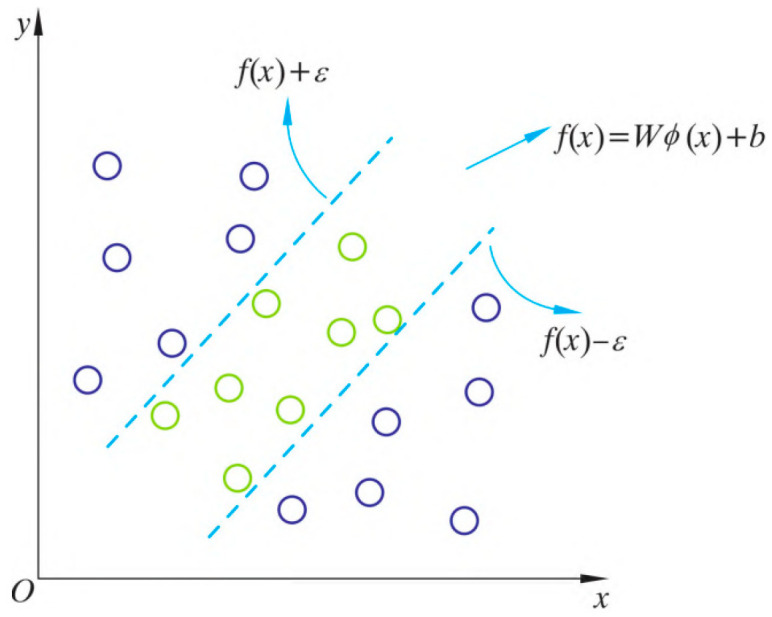
Schematic Diagram of Support Vector Machine Principle.

Vapnik (1995) [12] introduced the support vector machine (SVM) within the framework of statistical learning theory, where the principle of structural risk minimization ensures strong generalization performance. When SVM is applied to regression by incorporating an ε-insensitive loss function, it yields the support vector regression (SVR) model, which effectively addresses small-sample settings, pronounced nonlinearity, high dimensionality, and local minima. SVR has long been a focal topic in artificial intelligence and machine learning and is widely employed in fields such as engineering and economics. The fundamental procinple schematic diagram is shown in Figure 8.

The SVR model is essentially a constrained optimization problem that can provide a globally optimal solution. Its basic principle is as follows: when dealing with nonlinear problems, samples can be mapped from the original input space to a higher-dimensional feature space, where the data becomes more linearly separable. This mapping is achieved through a nonlinear function, known as the feature mapping function. If we denote the samples in the feature space as:

(25) $$\left(\phi (x_{1}),y_{1}),(\phi (x_{2}),y_{2}),\ldots ,(\phi (x_{n}),y_{n}\right)$$

Therefore, our goal is to find a hyperplane that separates these samples. The hyperplane can be represented as:

(26) ω⋅x+b=0

Here, ω is the normal vector of the hyperplane, and b is the intercept. For any sample (x,y), the following holds:

(27) $$\left(\omega \cdot x_{i}+b\right)\cdot y_{i}>0$$

To find the optimal hyperplane, we first need to define a loss function L(ω,b), which measures the degree of classification error. A common loss function is the hinge loss:

(28) $$L(\omega ,b)=\frac{1}{n}\sum_{i=1}^{n}\max(0,1-y_{i}(\omega \cdot \Phi (x_{i})+b))+\frac{\lambda }{2}\|\omega {\|}^{2}$$

Among them, the first term represents the classification error, and the second term is the regularization term, where λ is the regularization coefficient.

The objective is to minimize the above loss function. For convex optimization problems, we can use Lagrangian duality theory to transform it into a dual problem. The optimal solution of the dual problem can be expressed as follows:

(29) $$\sum_{i=1}^{n}\alpha_{i}-\frac{1}{2}\sum_{i=1}^{n}\sum_{j=1}^{n}\alpha_{i}\alpha_{j}y_{i}y_{j}\phi (x_{i})\cdot \phi (x_{j})$$

Among them, $\alpha_{i}$ is the Lagrange multiplier. In practical computations, the feature mapping function calculates the inner product through the kernel function $\phi (x_{i})\cdot \phi (x_{j})$.

The kernel function selected in this paper is the Gaussian kernel function, which is expressed as:

(30) $$K(x_{i},x_{j})=\exp\left(-\frac{|x_{i}-x_{j}{|}^{2}}{2\sigma^{2}}\right)$$

Among them, σ denotes the width parameter of the Gaussian kernel function. The solution of SVM can be transformed into a convex quadratic programming problem. Specifically, for a sample set, the objective function of SVM can be expressed as:

(31) $$\underset{\omega ,b,\xi }{\min}\frac{1}{2}\|\omega {\|}^{2}+C\sum_{i=1}^{m}\xi_{i}$$

Among them, ω is the normal vector of the hyperplane, b is the intercept of the hyperplane, ξ is the slack variable, C is the regularization parameter, and *m* is the number of samples.

The constraints of SVM can be expressed as:

(32) $$y_{i}(\omega^{T}x_{i}+b)\geq 1-\xi_{i},\xi_{i}\geq 0,i=1,\ldots ,m$$

By solving the above convex quadratic programming problem, the normal vector and intercept of the hyperplane can be obtained, thereby completing the regression task for the samples.

### 3.3. IBKA-SVR Model

The IBKA algorithm is employed to optimize the hyperparameters of the SVR model, thereby enabling the prediction of residential building energy consumption. Hence, the prediction process of the IBKA-SVR model is as follows:

Step 1: Standardize the experimental dataset. Sample data are obtained through numerical simulation or experiments and then standardized to construct the experimental dataset. To enhance the generalization ability of the model, the samples are reordered and subsequently divided into a training set and a test set based on their size, with the test set accounting for 30%.

Step 2: Parameter setting. This includes IBKA parameters such as maximum number of iterations, population size, and variable dimension d, as well as the parameter range of SVR.

Step 3: Initialize the population sizepop. The population must be initialized before obtaining the optimal parameter set of SVR. Here, it is assumed that the population is randomly distributed throughout the entire search space.

Step 4: Search for new solutions. New solutions are generated by updating using the IBKA formulas described in Section 2.1 and Section 2.2.

Step 5: Fitness evaluation. The mean squared error (MSE) is selected as the fitness function. The value of this function is obtained using the k-fold cross-validation (k-CV) method. The k-CV approach effectively mitigates overfitting.

**Figure 9:**
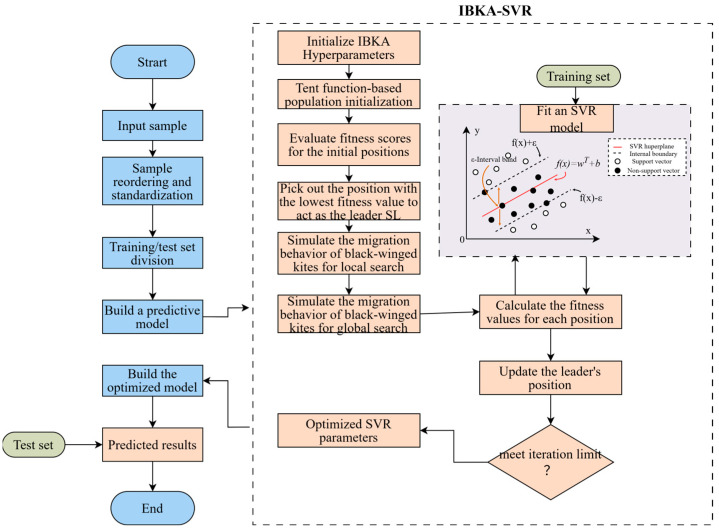
IBKA-SVR flow.

Step 6: Termination criterion. If the termination condition is met, output the result; otherwise, repeat Steps 4 and 5 in a loop until the preset number of iterations is reached, while ensuring convergence of the corresponding fitness function. This yields the optimal SVR parameter set, based on which the SVR model is trained using the training set and subsequently used to conduct prediction experiments on the test set. The corresponding IBKA-SVR flow is illustrated in Figure 9:

## 4. Energy Consumption Prediction Model for Residential Buildings

### 4.1. Residential Building Model

**Figure 10:**
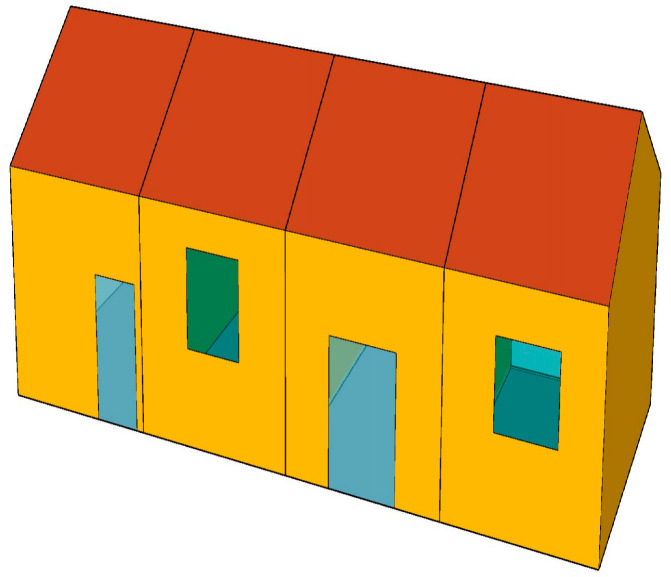
Three-dimensional model of residential buildings.

**Figure 11:**
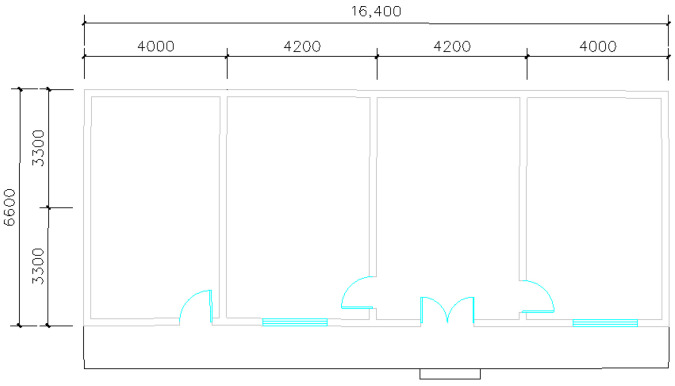
Baseline building plan of residential houses.

This study selected southern Shaanxi Province as the research area. Through field investigations and literature reviews, a typical representative type of residential building was chosen as the research object. The functional space layout, construction methods, and building materials of this building are characteristic and representative. Its interior structure and building area are also typical of the region, an adiabatic boundary is assumed to exist between the building floor slab and the ground. The building has an east–west width of 16.4 m, a north–south depth of 6.6 m, and a story height of 3.9 m. It features a gable roof and a total floor area of 103.3 m^2^. The occupancy schedule, set within the “Activity Template,” reflects the routines of local farmers in Southern Shaanxi, whose primary work is agriculture. Consequently, the building has low daytime occupancy and high nighttime occupancy. The metabolic rate was set to 0.9 met, and the winter clothing insulation was set to 1.7 clo (1 clo = 1Clo = 0.155 m^2^·K/W). The three-dimensional model of the building is shown in Figure 10, and its base plan is illustrated in Figure 11. The residential building model was established using TRNSYS software.

### 4.2. Initial Parameter Settings

#### 4.2.1. Envelope Structure Parameter Settings

According to the provisions on building heat transfer coefficient limits in the “Code for Thermal Design of Civil Buildings” (GB50176-2016) issued by the Ministry of Housing and Urban-Rural Development, this paper sets the initial parameters of the model as shown in Table 4.

#### 4.2.2. Simulation Parameter Settings

The activity schedules were set according to the daily routines of rural residents in southern Shaanxi Province. As farming is the primary livelihood for local farmers, indoor occupancy is relatively low during daytime hours and significantly higher in the evenings. The indoor heating design temperature was set at 18 °C, with an air change rate of 1 time per hour. The average PMV value over a three-month heating period was selected as the simulation output for indoor thermal comfort. The PMV simulation parameter settings are shown in the Table 5.

### 4.3. Simulation Results of Typical Residential Buildings

**Figure 12:**
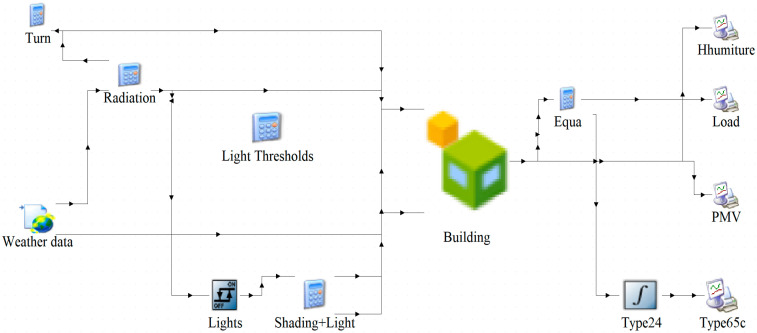
Residential Simulation Model.

A residential building simulation model was established using TRNSYS, as shown in Figure 12.

### 4.4. Selection and Values of Energy Consumption Impact Factors

This paper mainly discusses the impacts of changing the window-wall ratio (adjusting the window area), altering the insulation layer thickness (adding insulating materials to the building envelope and varying the insulation thickness for experiments), and incorporating a sunspace (adding a sunroom and varying its depth for experiments) on the modeling outcomes. The selected impact factors include Window-wall Ratio in the East, Window-wall Ratio in the West, Window-wall Ratio in the South, Window-wall Ratio in the North, Sunspace Depth, Insulation Thickness in Outer Wall, and Insulation Thickness in Roof Covering. Each factor includes seven levels. The selection and values of these energy consumption impact factors are shown in Table 6.

### 4.5. Energy Consumption Analysis Results

This study analyzed seven factors, each at seven distinct levels (Levels 1–7, as detailed in Table 6): Window-wall Ratio (WWR) for the four cardinal directions (East, West, South, North), Sunspace Depth, Outer Wall Insulation Thickness, and Roof Insulation Thickness. Using SPSS 23, an L49(7^8^) standard orthogonal array was employed to structure the experiments. After removing superfluous columns, this design yielded 49 unique schemes. Using TRNSYS 17, simulation models corresponding to these 49 combinations were established to perform energy consumption simulations under different factor combinations. The energy consumption data for each of the 49 combinations were thereby obtained. The 49 schemes and the corresponding energy consumption simulation results are summarized in Table 7.

### 4.6. Performance of the Residential Building Energy Consumption Prediction Model

**Figure 13:**
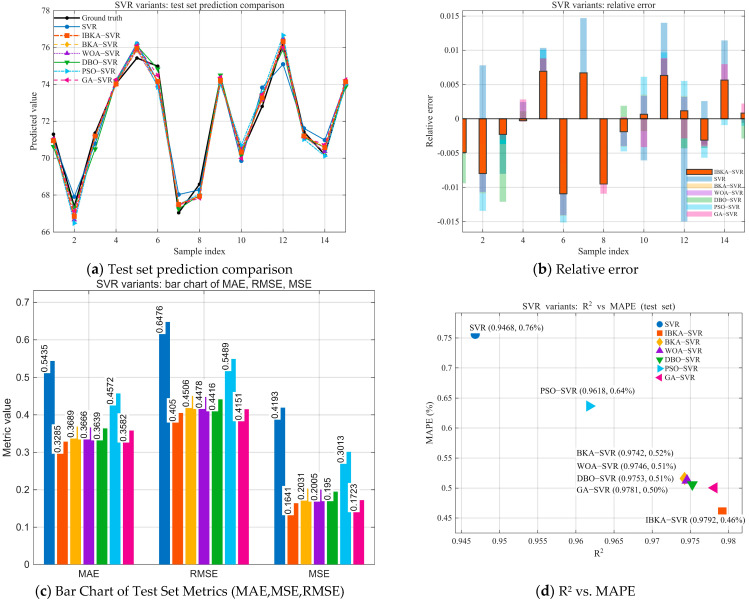
Evaluation Index of SVR and Six Optimized Models for Energy Consumption Prediction.

To verify the effectiveness of the improved BKA (IBKA) algorithm for optimizing SVR hyperparameters (C, γ, ε) and improving prediction accuracy, we performed numerical simulations in MATLAB 2025a. The dataset of 49 samples was split into training (70%) and testing (30%) sets, with 5-fold cross-validation applied to the training set during the optimization process to ensure generalization [5]. We compared our proposed IBKA-SVR model against two types of benchmarks: (1) a standard SVR model without optimization, and (2) SVR models optimized by five other metaheuristic algorithms (BKA, WOA, DBO, PSO, and GA). Performance was evaluated using five key metrics (MSE, RMSE, MAE, MAPE, and R^2^), with the results summarized in Table 8 and Figure 13.

The results presented in Table 8 and Figure 13 indicate that the IBKA-SVR model achieves significant improvements across all evaluation metrics. Compared to the unoptimized SVR, IBKA-SVR reduces MAE from 0.5435 to 0.3285 (a decrease of ~39.6%), MAPE from 0.7561 to 0.4606 (~39.1%), RMSE from 0.6476 to 0.405 (~37.5%), and MSE from 0.4193 to 0.1641 (~60.9%), while R^2^ increases from 0.9468 to 0.9792 (an absolute improvement of 3.24 percentage points). This demonstrates that IBKA can effectively enhance the fitting accuracy and generalization ability of SVR. Furthermore, IBKA-SVR maintains its overall superiority when compared to the best-performing models optimized by other typical algorithms. In comparison to GA-SVR (MAE = 0.3582, RMSE = 0.4151, MSE = 0.1723, R^2^ = 0.9781), IBKA-SVR further decreases MAE and MAPE by approximately 8.3% and 8.0%, and RMSE and MSE by approximately 2.4% and 4.8%, respectively, while increasing R^2^ by another 0.11 percentage points. Compared to DBO-SVR and WOA-SVR, IBKA-SVR still achieves reductions of about 8–18% in error-based metrics (RMSE, MSE). Relative to PSO-SVR, IBKA-SVR generally obtains improvements of approximately 26–46% across all error metrics, accompanied by a higher R^2^.

In summary, by leveraging a more efficient global search and synergistic parameter optimization, IBKA achieves a better solution in the bias/variance trade-off. This enables the SVR model to exhibit the strongest comprehensive advantage among the five-benchmark optimizer and the baseline model.

### 4.7. SHAP-Based Model Interpretation

The SHAP (Shapley Additive exPlanations) model is an additive feature attribution method based on cooperative game theory [27]. It serves as a framework for explaining the predictions of machine learning models by quantifying the impact of each feature on the model’s decision. This is achieved by fairly allocating the marginal contribution of feature subsets to the prediction outcome. The contribution of each input feature is represented by its Shapley value. To enhance interpretability, SHAP approximates a black-box model f(x) by constructing a linear explanation model g(x′), whose formula is

(33) $$f(x)=g(x\prime )=\phi_{0}+\sum_{i=1}^{P}\phi_{i}x_{i}^{\prime }$$

In this formula, $\phi_{0}$ represents the mean prediction over all samples, $\phi_{i}$ is the SHAP value of feature i, and $x_{i}^{\prime }$ is the simplified feature representation, which takes a value of either 0 (indicating the feature is absent) or 1 (indicating it is present). P is the number of input features.

The SHAP value $\phi_{i}$ for feature i is calculated by the following formula:

(34) $$\phi_{i}(f,x)=\sum_{S\subseteq F\left\{i\right\}}\frac{|S|\cdot (|F|-|S|-1)!}{|F|!}\left[f_{S\cup \left\{i\right\}}(x_{S\cup \left\{i\right\}})-f_{S}(x_{S})\right]$$

In this formula, S represents a subset of features, F denotes the set of all features, and $f_{S}$ is the model’s output for the subset S.

#### 4.7.1. Feature Importance Analysis

**Figure 14:**
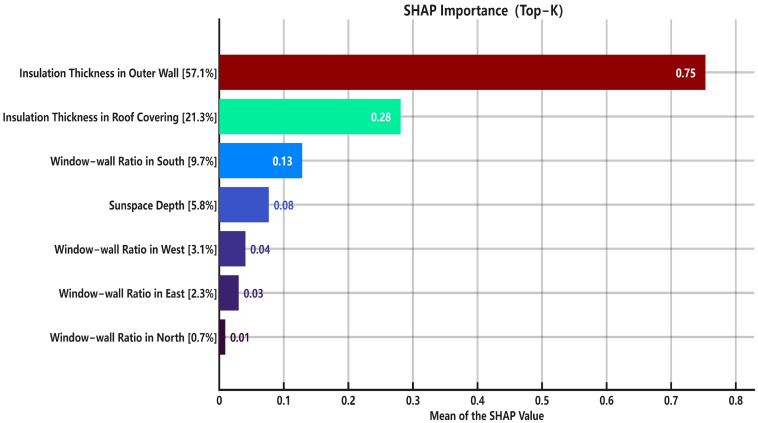
Feature importance Ranking.

Figure 14 presents the feature importance ranking as calculated by SHAP. The analysis reveals that, quantitatively, the most significant factor is Insulation Thickness in Outer Wall (57.1%). This is followed, in descending order of importance, by Insulation Thickness in Roof Covering (21.3%), Window-wall Ratio in the South (9.7%), Sun space Depth (5.8%), Window-wall Ratio in the West (3.1%), Window-wall Ratio in the East (2.3%), and Window-wall Ratio in the North (0.7%).

#### 4.7.2. Analysis of Feature Impact Distribution

**Figure 15:**
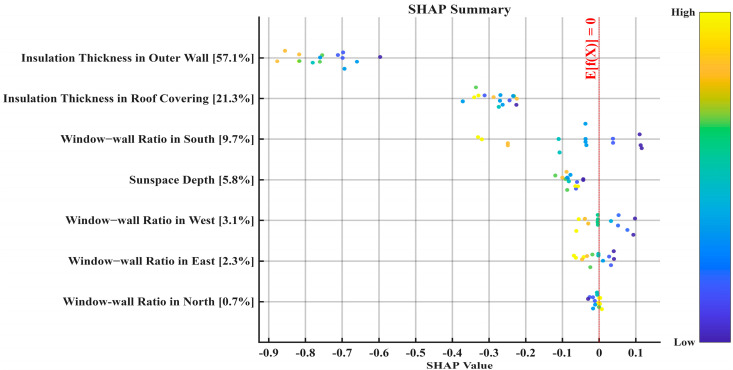
Feature Influences Distribution.

Figure 15 displays the SHAP summary plot, which illustrates the distribution of feature impacts. On this plot, the horizontal axis represents the SHAP value for each data point, while the vertical axis lists the features, ranked from top to bottom by their overall importance. The color of each point indicates the feature’s value for that instance, with yellow representing higher values and blue representing lower values. Overlapping points signify a higher density of data in that region. Therefore, by observing the distribution of points and their color variations, we can determine the direction and magnitude of each feature’s impact on the Civil Residential Building Energy Consumption. The analysis of Figure 6 reveals the following: Insulation Thickness in Outer Wall and Insulation Thickness in Roof Covering have the most significant impact. For Insulation Thickness in Outer Wall, as the feature value increases, the Shapley value shows a decreasing trend. This indicates a strong, non-linear negative correlation with the prediction outcome. For Insulation Thickness in Roof Covering and Sun space Depth, the interspersion of yellow and blue points suggests a complex, non-linear relationship. The Window-wall Ratio in the North exhibits a positive correlation. For the three features, Window-wall Ratio in the South, Window-wall Ratio in the West, and Window-wall Ratio in the East, the yellow and blue points are intermingled on both sides of the zero-SHAP-value axis. Consequently, a more detailed analysis of these variables requires examining individual feature dependence plots and their specific values.

#### 4.7.3. Single-Feature Dependence Analysis

**Figure 16:**
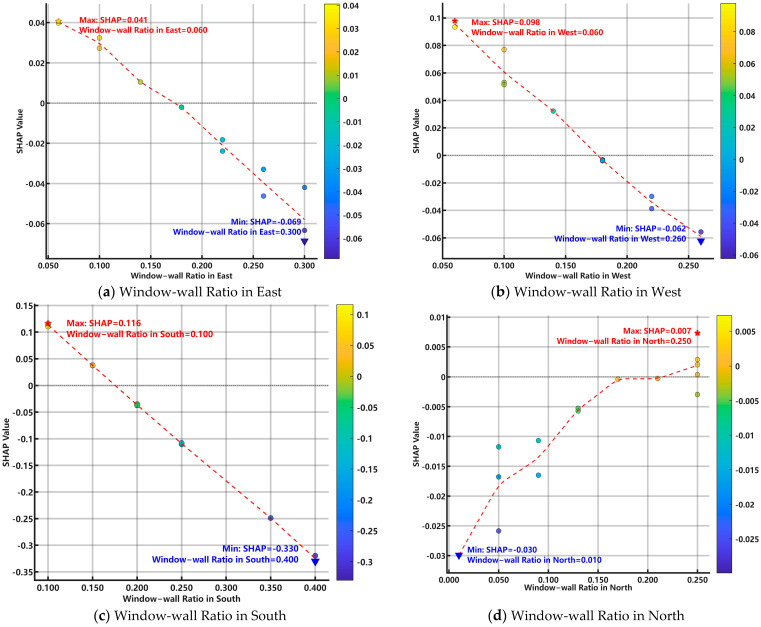

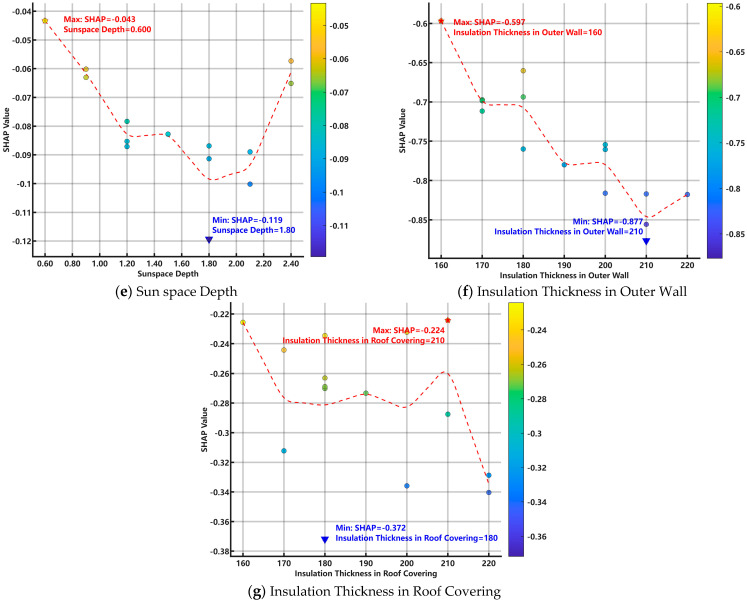
Feature dependence plots.

Figure 16 displays seven feature dependence plots. These sample scatter plots enable an analysis of how the value of each feature influences Residential Building Energy Consumption and reveal the patterns governing this relationship.

An analysis of the individual feature dependence plots reveals the following relationships:

Insulation Thickness in Outer Wall: With SHAP values ranging from −0.877 to −0.597, this feature has a significant negative impact. As the insulation thickness increases, the SHAP value consistently decreases, indicating a strong, non-linear negative correlation with the predicted energy consumption. Insulation Thickness in Roof Covering: SHAP values range from −0.372 to −0.224, indicating a significant negative impact. Similar to the outer wall, increasing roof insulation thickness leads to a decrease in the SHAP value in a non-linear fashion. Window-wall Ratio in the South: With a SHAP value range of −0.330 to 0.116, this feature has a large negative impact. The relationship is approximately linear, as higher ratios tend to decrease the predicted energy consumption. Window-wall Ratio in the West: SHAP values range from −0.062 to 0.098, showing a large negative impact. The relationship is nearly linear, with SHAP values monotonically decreasing as the feature value increases. Window-wall Ratio in the East: With a SHAP value range of −0.069 to 0.041, this feature has a moderate negative impact. The relationship is also nearly linear and negative. Sun space Depth: SHAP values range from −0.119 to 0.043, indicating a moderate and complex impact. As the depth increases, the SHAP value first decreases and then increases, showing a non-linear relationship where the influence shifts from negative to positive. Window-wall Ratio in the North: With a SHAP value range of −0.030 to 0.007, this feature has a minor positive impact. As the ratio increases, the SHAP value shows a slight upward trend, indicating a non-linear positive correlation.

In summary, the descending order of feature influence is as follows: Insulation Thickness in Outer Wall > Insulation Thickness in Roof Covering > Window-wall Ratio in the South > Window-wall Ratio in the West > Window-wall Ratio in the East > Sun space Depth > Window-wall Ratio in the North.

#### 4.7.4. Analysis of Feature Interaction Effects

**Figure 17:**
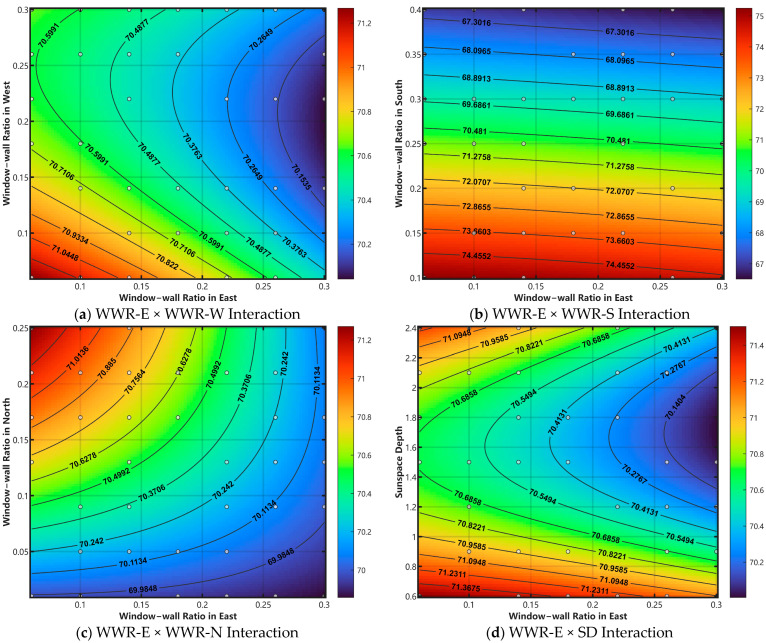

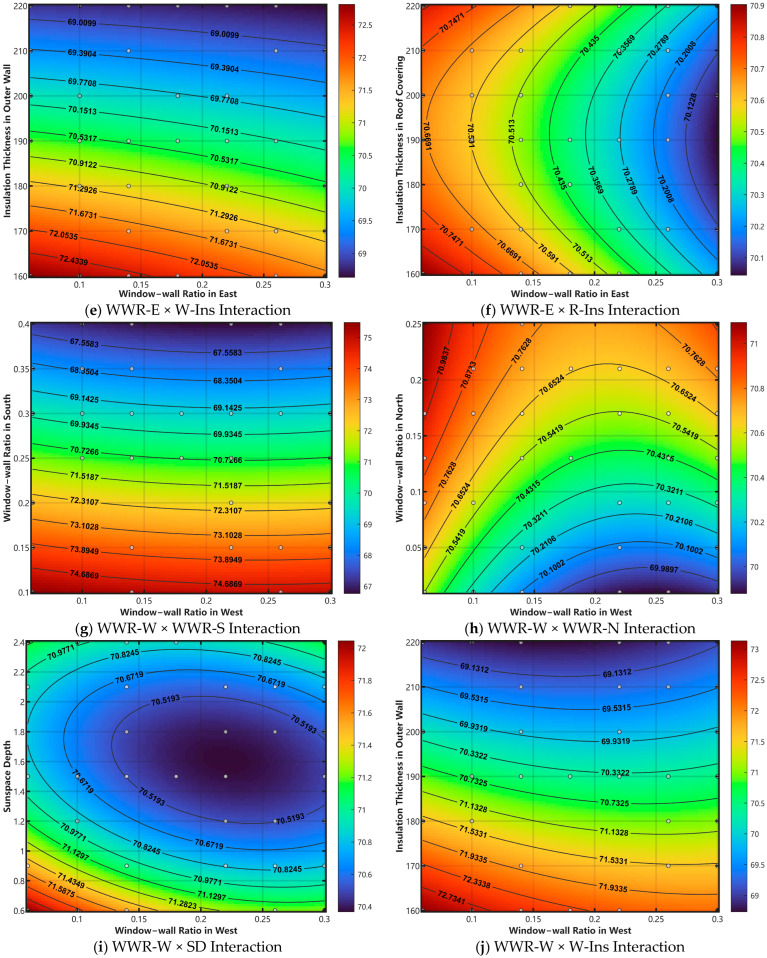

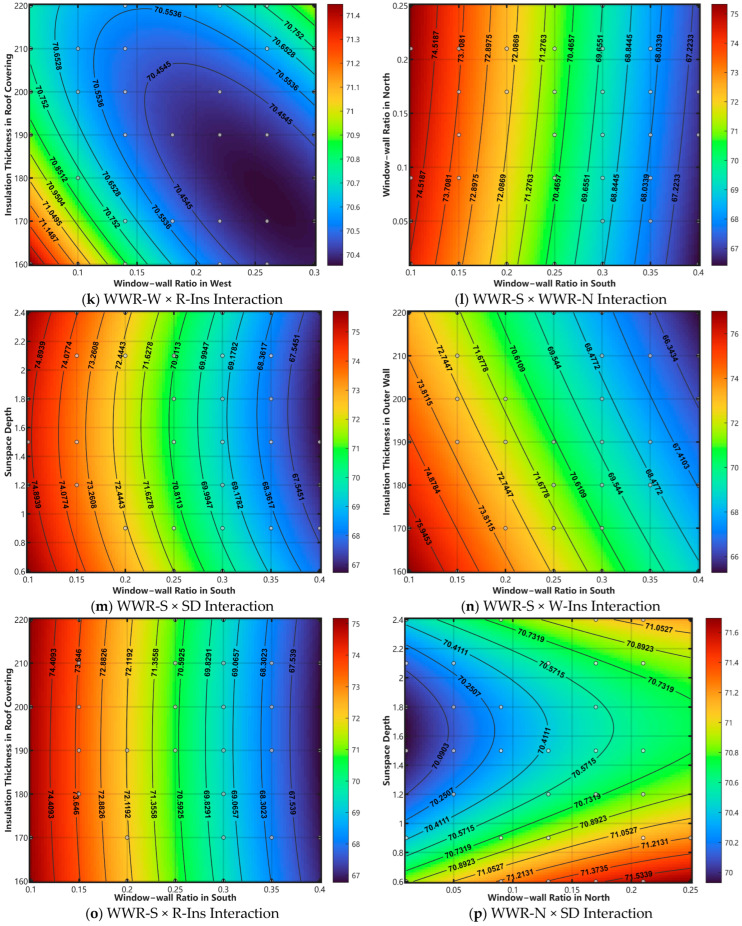

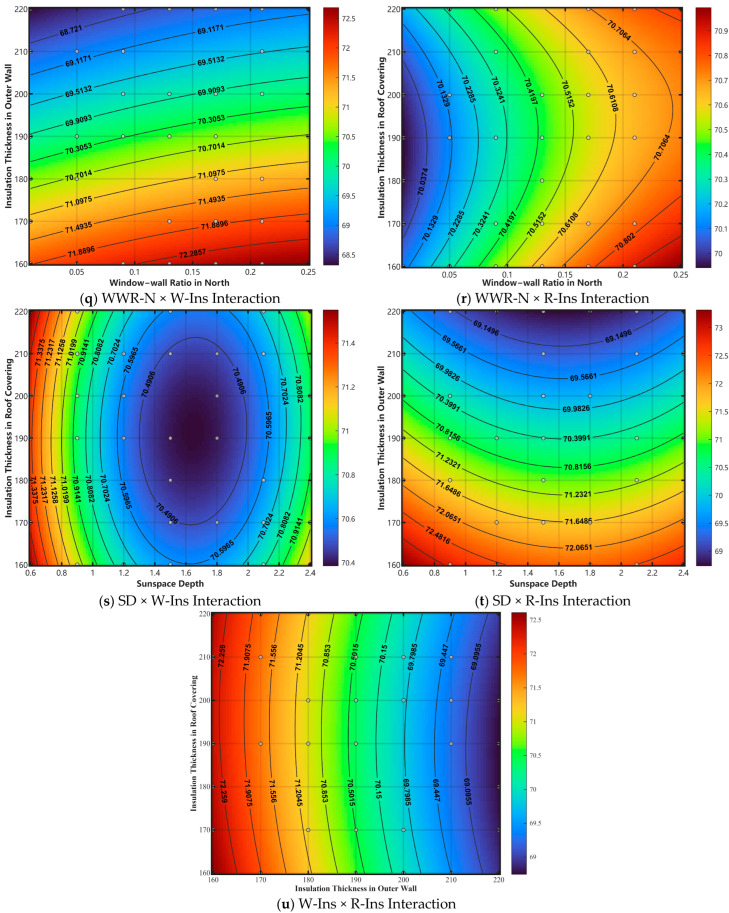
Contour plots.

Figure 17 presents a series of contour plots illustrating the interaction effects among seven features: Window-wall Ratio in the East (WWR-E), Window-wall Ratio in the West (WWR-W), Window-wall Ratio in the South (WWR-S), Window-wall Ratio in the North (WWR-N), Sun space Depth (SD), Insulation Thickness in Outer Wall (W-Ins), and Insulation Thickness in Roof Covering (R-Ins). These plots enable an analysis of how the combined values of these features influence Residential Building Energy Consumption and reveal the patterns of their interactive impact.

The analysis of the contour plots reveals a clear hierarchy of interaction effects: Strong to Very Strong Interactions: A significant number of feature pairs exhibit strong coupling. These include plots (a), (c), (d), (f), (h), (i), (k), (p), and (t). Their contours are characterized by significant arc-shapes, non-parallel curves, valley/ridge formations, or closed concentric ellipses. This indicates that the effect or optimal value of one variable is highly dependent on the value of the other. The interactions in plots (h), (i), and (t) are particularly strong, suggesting the variables are deeply coupled.

Moderate Interactions: Plots (j), (r), and (s) show moderate interaction effects. Their contours, often “U”-shaped or smooth arcs, indicate that while the variables are not fully independent, their optimal ranges shift in a predictable but non-linear way relative to each other.

Weak to No Interactions: The remaining plots show weak, very weak, or virtually no interaction effects. These include plots (b), (e), (g), (l), (m), (n), (o), (q), and (u). Their contours are predominantly parallel straight lines (whether slanted, horizontal, or vertical) or only slightly curved. This demonstrates that the influence of one variable on the outcome is largely independent of the other variable’s value in the pair.

Summary of Findings: The interaction analysis reveals significant differences in interaction strengths among the design variables, forming a clear hierarchy. The strongest interactions are observed among Sun space Depth (SD), Insulation Thickness in Roof Covering (R-Ins), and the Window-wall Ratios for the East, West, and North facades (WWR-E, WWR-W, WWR-N). Their contour plots, characterized by closed ellipses or significantly distorted curves, indicate that the effects of these variables are deeply coupled. Conversely, the Window-wall Ratio in the South (WWR-S) exhibits very weak or negligible interaction with almost all other variables.

## 5. Conclusions

To address the issues of rudimentary construction, poor thermal performance of envelope structures, and high energy consumption in Chinese residential buildings, this study analyzed the factors influencing building energy consumption. An improved BKA algorithm is proposed to optimize the support vector regression machine for establishing a residential building energy consumption prediction and analysis model. This method introduces several improvement strategies into the BKA algorithm, including randomly selecting attack behaviors, incorporating global information and randomness into position updates, and applying NGO random selection operators. The IBKA algorithm is used to optimize the parameters of the SVR-based residential building energy consumption prediction and analysis model. Finally, to elucidate the model’s internal decision-making mechanism, the SHAP (SHapley Additive exPlanations) interpretability framework was employed to quantitatively clarify the impact of both the independent contributions and the interaction effects of each influencing factor on energy consumption.

(1) The effectiveness of the IBKA algorithm was verified using the CEC2017 benchmark functions. Compared with the BKA and six other algorithms, the improved BKA algorithm demonstrates enhanced initial population diversity, convergence accuracy, and global search capability.

(2) By employing the proposed IBKA algorithm for hyperparameter optimization, the Support Vector Regression (SVR) model demonstrated exceptional predictive accuracy and generalization capability. Its performance is not only markedly superior to the baseline model, achieving a substantial reduction of 37–60% in key error metrics, but it also comprehensively outperforms SVR models tuned by five other representative optimizers, including BKA, GA, and PSO, across all evaluation metrics.

(3) The ranking of the design parameters’ impact on residential building energy consumption, from greatest to least, is as follows: Insulation Thickness in Outer Wall > Insulation Thickness in Roof Covering > Window-wall Ratio in the South > Window-wall Ratio in the West > Window-wall Ratio in the East > Sun space Depth > Window-wall Ratio in the North. The analysis reveals that the Insulation Thickness in Outer Wall and Insulation Thickness in Roof Covering are the dominant factors, both exhibiting a strong, non-linear negative correlation with energy consumption. The Window-wall Ratios in the South, West, and East are secondary factors, demonstrating an approximately linear and negative relationship. In contrast, the Sun space Depth and the Window-wall Ratio in the North display more complex, non-linear relationships, with the latter being the sole parameter to exhibit a positive correlation.

(4) The strongest interaction effects are observed among Sun space Depth (SD), Insulation Thickness in Roof Covering (R-Ins), and the Window-wall Ratios in the East (WWR-E), West (WWR-W), and North (WWR-N). This is visually evidenced in their contour plots, which display closed elliptical shapes or significantly distorted curves, indicating that the effects of these variables are strongly coupled and interdependent. In stark contrast, the Window-wall Ratio in the South (WWR-S) exhibits negligible or very weak interaction effects with nearly all other parameters.

In conclusion, this study has successfully developed and validated an integrated bio-inspired modeling and analysis framework, which combines the improved Black-winged Kite Optimization Algorithm (IBKA), Support Vector Regression (SVR), and SHAP for explainability. The results demonstrate that this framework not only effectively predicts and analyzes residential building energy consumption with high precision, but more importantly, it transcends being a mere predictive tool to become a comprehensive decision-support system. By simultaneously providing accurate predictions and transparent, actionable insights into how design variables interact, it empowers architects and policymakers to optimize new building designs, prioritize the most effective retrofitting strategies, and formulate data-driven energy policies. This directly translates the model’s analytical power into tangible contributions toward achieving energy efficiency and sustainability in rural residential buildings.

## 6. Limitations and Future Work

While the proposed IBKA-SVR model has demonstrated strong performance and provided valuable insights, this study has several limitations that open avenues for future research.

### 6.1. Limitations of the Study

1. Simplifications in the Physical Model: To maintain a focus on the key features of the building envelope, several simplifications were made to the physical model. Specifically, the model omitted a detailed foundation and soil model, thereby neglecting the significant impact of factors like soil type and moisture content on ground-coupled heat transfer. Furthermore, we did not conduct a dedicated sensitivity analysis on errors associated with initial boundary conditions, such as ground and envelope temperatures. Finally, the study is centered on static building features, and does not yet incorporate dynamic variables such as occupant behaviors, which are critical determinants of actual energy consumption.

2. Data Source, Scale, and Model Validation: The foremost limitation of this study is its reliance on a simulation-generated dataset of a relatively limited scale (Sample Numbers = 49), coupled with the absence of empirical data from real-world, monitored buildings. Although the simulation engine is well-validated and the input parameters conform to local standards, the simulation model itself has not undergone formal calibration against empirical data. This may introduce discrepancies between simulated outcomes and actual energy performance. Moreover, the small dataset size constrains the feasibility of exploring more complex data-driven models, such as deep neural networks.

3. Specificity and Generalizability of the Study Scope: The current research is a case study focused on rural residences within a specific climate zone in China (Shaanxi Province). Consequently, the findings and parameter sensitivities may exhibit regional and climatic specificity. The direct extrapolation of these conclusions to other climate zones or different building typologies (e.g., commercial, agricultural, or mixed-use buildings) should be approached with caution, as its validity has not been established for these contexts.

4. Absence of Uncertainty Quantification: The study did not perform uncertainty quantification (UQ) for its predictions, such as providing confidence or prediction intervals. This omission is primarily attributed to the limited dataset size. The lack of uncertainty assessment curtails the model’s utility in providing robust support for risk-informed decision-making.

### 6.2. Future Research Directions

Based on the aforementioned limitations, we have outlined several key directions for future research to deepen and extend the contributions of this work:

1. Model Validation and Calibration with Empirical Data: The highest priority for future work involves conducting field studies to collect architectural plans and long-term energy consumption data from real, monitored rural residences. This empirical data will be used to perform a rigorous calibration and validation of both the TRNSYS 17 simulation and the IBKA-SVR prediction models. This will enhance their absolute predictive accuracy, test their generalizability on an independent real-world dataset, and ultimately bridge the gap between simulation and reality.

2. Dataset Expansion and Model Deepening: We plan to significantly expand the dataset by increasing the number of simulation runs and extending the scope to encompass other typical climate zones across China. Future research will also focus on developing more sophisticated physical models that incorporate dynamic occupant behavior patterns and detailed ground-coupled heat transfer models. A larger and more diverse dataset will enable the exploration of more advanced deep learning architectures and bolster the model’s robustness and generalizability.

3. Deepening Methodology and Uncertainty Analysis: We plan to conduct cross-validation using other mainstream building energy simulation software (e.g., EnergyPlus 23.2.0, IES VE 2023). Methodologically, future research could implement techniques such as bootstrapping or Bayesian neural networks to quantify prediction uncertainty. Furthermore, we will actively explore cutting-edge approaches like Physics-Informed Neural Networks (PINNs) to integrate physical governing equations with data-driven models, aiming to enhance predictive accuracy, particularly in low-data regimes.

4. Promoting Practical Application and System Integration: We will actively seek collaborations with building designers, engineers, and policymakers to facilitate the practical translation of our research findings. The long-term vision is to integrate the developed high-fidelity IBKA-SVR model as a module within Building Energy Management Systems (BEMS) or early-stage design tools like Auto CAD 2023 software. This would provide stakeholders with a powerful tool for real-time optimization and decision support for both new low-energy building designs and the retrofitting of existing buildings.

## Figures and Tables

**Table 1:** Ablation Study Results.

Variant	AvgRank	Wins	Ties	Losses	Ps_mean_	ERTi_mean_	ERTe_mean_
Base	5.571	0	14	0	0.267	1632	97,976
+Imp1	5.929	5	0	9	0.205	1259	75,597
+Imp2	5.214	7	0	7	0.269	65,535	65,535
+Imp3	4.214	11	0	3	0.376	1276	76,572
+Imp4	6.143	5	0	9	0.169	1646	98,813
+Imp1+Imp2	4.500	9	0	5	0.288	1818	109,120
+Imp1+Imp2+Imp3	2.929	13	0	1	0.590	1403	84,204
+Imp1+Imp2+Imp3+Imp4	1.500	14	0	0	0.664	1018	61,109

**Table 2:** Testing Results of Eight Algorithms.

Function	Metric	IBKA	BKA	GJO	DBO	WOA	KOA	HPO	SABO
F1	std	6.77 × 10^0^	1.43 × 10^10^	4.20 × 10^9^	1.86 × 10^6^	2.88 × 10^7^	9.43 × 10^7^	6.87 × 10^3^	2.51 × 10^9^
	avg	1.04 × 10^2^	6.49 × 10^9^	1.08 × 1010	6.20 × 10^5^	3.02 × 10^7^	2.40 × 10^8^	8.60 × 10^3^	6.52 × 10^9^
	median	1.02 × 10^2^	3.56 × 10^8^	9.68 × 10^9^	2.44 × 10^4^	2.09 × 10^7^	2.23 × 10^8^	5.59 × 10^3^	6.07 × 10^9^
	*p*-value	-	3.02 × 10^−11^	3.02 × 10^−11^	3.02 × 10^−11^	3.02 × 10^−11^	3.02 × 10^−11^	3.02 × 10^−11^	3.02 × 10^−11^
F3	std	2.34 × 10^−13^	2.93 × 10^4^	7.99 × 10^3^	1.52 × 10^4^	6.53 × 10^4^	2.28 × 10^4^	3.34 × 10^3^	7.92 × 10^3^
	avg	3.00 × 10^2^	1.90 × 10^4^	4.59 × 10^4^	2.40 × 10^4^	1.75 × 10^5^	1.20 × 10^5^	2.70 × 10^3^	3.97 × 10^4^
	median	3.00 × 10^2^	6.70 × 10^3^	4.63 × 10^4^	2.04 × 10^4^	1.63 × 10^5^	1.19 × 10^5^	8.08 × 10^2^	4.04 × 10^4^
	*p*-value	-	2.65 × 10^−11^	2.65 × 10^−11^	2.65 × 10^−11^	2.65 × 10^−11^	2.65 × 10^−11^	2.65 × 10^−11^	2.65 × 10^−11^
F4	std	3.10 × 10^1^	3.02 × 10^3^	1.08 × 10^3^	4.40 × 10^1^	3.79 × 10^1^	4.84 × 10^1^	2.84 × 10^1^	8.85 × 10^2^
	avg	4.60 × 10^2^	1.54 × 10^3^	1.42 × 10^3^	5.28 × 10^2^	5.61 × 10^2^	6.08 × 10^2^	4.77 × 10^2^	1.47 × 10^3^
	median	4.71 × 10^2^	5.39 × 10^2^	1.11 × 10^3^	5.19 × 10^2^	5.64 × 10^2^	5.99 × 10^2^	4.80 × 10^2^	1.12 × 10^3^
	*p*-value	-	6.72 × 10^−10^	3.02 × 10^−11^	2.92 × 10^−9^	4.50 × 10^−11^	3.02 × 10^−11^	6.79 × 10^−2^	3.02 × 10^−11^
F5	std	4.43 × 10^1^	4.75 × 10^1^	2.65 × 10^1^	5.50 × 10^1^	4.66 × 10^1^	2.17 × 10^1^	3.56 × 10^1^	2.70 × 10^1^
	avg	7.11 × 10^2^	7.37 × 10^2^	6.88 × 10^2^	7.35 × 10^2^	8.20 × 10^2^	7.43 × 10^2^	6.79 × 10^2^	7.75 × 10^2^
	median	7.02 × 10^2^	7.30 × 10^2^	6.86 × 10^2^	7.37 × 10^2^	8.09 × 10^2^	7.43 × 10^2^	6.84 × 10^2^	7.76 × 10^2^
	*p*-value	-	4.68 × 10^−2^	1.84 × 10^−2^	4.68 × 10^−2^	5.07 × 10^−10^	3.67 × 10^−3^	5.83 × 10^−3^	1.16 × 10^−7^
F6	std	1.22 × 10^1^	8.79 × 10^0^	8.76 × 10^0^	1.45 × 10^1^	8.16 × 10^0^	5.99 × 10^0^	9.44 × 10^0^	1.10 × 10^1^
	avg	6.42 × 10^2^	6.62 × 10^2^	6.32 × 10^2^	6.47 × 10^2^	6.70 × 10^2^	6.19 × 10^2^	6.25 × 10^2^	6.54 × 10^2^
	median	6.41 × 10^2^	6.61 × 10^2^	6.31 × 10^2^	6.49 × 10^2^	6.69 × 10^2^	6.18 × 10^2^	6.23 × 10^2^	6.52 × 10^2^
	*p*-value	-	4.69 × 10^−8^	1.86 × 10^−3^	7.98 × 10^−2^	2.61 × 10^−10^	3.16 × 10^−10^	1.11 × 10^−6^	5.26 × 10^−4^
F7	std	9.07 × 10^1^	7.80 × 10^1^	5.68 × 10^1^	8.36 × 10^1^	9.10 × 10^1^	2.77 × 10^1^	6.35 × 10^1^	4.79 × 10^1^
	avg	1.08 × 10^3^	1.22 × 10^3^	1.02 × 10^3^	1.01 × 10^3^	1.23 × 10^3^	1.02 × 10^3^	1.02 × 10^3^	1.08 × 10^3^
	median	1.05 × 10^3^	1.22 × 10^3^	1.02 × 10^3^	1.01 × 10^3^	1.24 × 10^3^	1.02 × 10^3^	1.01 × 10^3^	1.08 × 10^3^
	*p*-value	-	3.01 × 10^−7^	6.97 × 10^−3^	6.10 × 10^−3^	2.15 × 10^−6^	1.00 × 10^−3^	2.27 × 10^−3^	4.83 × 10^−1^
F8	std	3.29 × 10^1^	5.74 × 10^1^	3.12 × 10^1^	4.53 × 10^1^	6.74 × 10^1^	2.66 × 10^1^	4.38 × 10^1^	3.65 × 10^1^
	avg	9.55 × 10^2^	9.87 × 10^2^	9.68 × 10^2^	1.01 × 10^3^	1.01 × 10^3^	1.04 × 10^3^	9.58 × 10^2^	1.06 × 10^3^
	median	9.50 × 10^2^	9.68 × 10^2^	9.65 × 10^2^	1.00 × 10^3^	9.89 × 10^2^	1.04 × 10^3^	9.50 × 10^2^	1.06 × 10^3^
	*p*-value	-	1.03 × 10^−2^	7.48 × 10^−2^	5.09 × 10^−6^	1.75 × 10^−5^	7.38 × 10^−10^	9.23 × 10^−1^	1.61 × 10^−10^
F9	std	6.95 × 10^2^	1.01 × 10^3^	8.12 × 10^2^	1.76 × 10^3^	3.55 × 10^3^	9.00 × 10^2^	1.20 × 10^3^	1.58 × 10^3^
	avg	3.92 × 10^3^	5.01 × 10^3^	3.90 × 10^3^	5.06 × 10^3^	8.76 × 10^3^	2.94 × 10^3^	5.10 × 10^3^	5.14 × 10^3^
	median	3.87 × 10^3^	4.84 × 10^3^	3.81 × 10^3^	4.42 × 10^3^	7.94 × 10^3^	2.79 × 10^3^	4.86 × 10^3^	4.78 × 10^3^
	*p*-value	-	5.60 × 10^−7^	8.65 × 10^−1^	7.29 × 10^−3^	1.46 × 10^−10^	3.16 × 10^−5^	2.96 × 10^−5^	1.78 × 10^−4^
F10	std	7.97 × 10^2^	9.33 × 10^2^	1.25 × 10^3^	9.61 × 10^2^	8.30 × 10^2^	4.13 × 10^2^	5.84 × 10^2^	3.91 × 10^2^
	avg	6.41 × 10^3^	5.38 × 10^3^	5.77 × 10^3^	6.17 × 10^3^	6.51 × 10^3^	8.56 × 10^3^	5.02 × 10^3^	8.37 × 10^3^
	median	6.49 × 10^3^	5.20 × 10^3^	5.64 × 10^3^	6.35 × 10^3^	6.41 × 10^3^	8.62 × 10^3^	4.85 × 10^3^	8.37 × 10^3^
	*p*-value	-	1.75 × 10^−5^	2.05 × 10^−3^	1.71 × 10^−1^	9.71 × 10^−1^	3.69 × 10^−11^	8.48 × 10^−9^	3.02 × 10^−11^
F11	std	5.49 × 10^1^	6.63 × 10^2^	1.36 × 10^3^	9.84 × 10^1^	8.64 × 10^2^	2.11 × 10^2^	8.82 × 10^1^	1.09 × 10^3^
	avg	1.25 × 10^3^	1.46 × 10^3^	3.11 × 10^3^	1.41 × 10^3^	2.14 × 10^3^	1.70 × 10^3^	1.29 × 10^3^	3.14 × 10^3^
	median	1.24 × 10^3^	1.30 × 10^3^	2.77 × 10^3^	1.40 × 10^3^	1.80 × 10^3^	1.64 × 10^3^	1.29 × 10^3^	2.93 × 10^3^
	*p*-value	-	9.79 × 10^−5^	3.02 × 10^−11^	5.97 × 10^−9^	3.34 × 10^−11^	3.02 × 10^−11^	5.37 × 10^−2^	3.02 × 10^−11^
F12	std	1.61 × 10^4^	2.63 × 10^9^	7.49 × 10^8^	1.62 × 10^7^	4.92 × 10^7^	9.27 × 10^6^	6.61 × 10^5^	6.64 × 10^8^
	avg	2.42 × 10^4^	9.39 × 10^8^	7.52 × 10^8^	1.34 × 10^7^	7.67 × 10^7^	1.39 × 10^7^	7.47 × 10^5^	3.64 × 10^8^
	median	2.03 × 10^4^	7.03 × 10^6^	4.27 × 10^8^	6.46 × 10^6^	6.97 × 10^7^	1.18 × 10^7^	5.38 × 10^5^	1.37 × 10^8^
	*p*-value	-	3.02 × 10^−11^	3.02 × 10^−11^	3.02 × 10^−11^	3.02 × 10^−11^	3.02 × 10^−11^	3.02 × 10^−11^	3.02 × 10^−11^
F13	std	1.34 × 10^4^	1.67 × 10^8^	1.25 × 10^8^	1.13 × 10^6^	8.91 × 10^4^	7.34 × 10^5^	2.60 × 10^4^	1.53 × 10^7^
	avg	1.11 × 10^4^	3.17 × 10^7^	1.39 × 10^8^	9.37 × 10^5^	1.42 × 10^5^	6.94 × 10^5^	3.76 × 10^4^	7.43 × 10^6^
	median	5.32 × 10^3^	1.56 × 10^5^	1.26 × 10^8^	2.14 × 10^5^	1.13 × 10^5^	3.84 × 10^5^	3.13 × 10^4^	1.93 × 10^6^
	*p*-value	-	3.69 × 10^−11^	4.50 × 10^−11^	3.69 × 10^−11^	4.98 × 10^−11^	3.02 × 10^−11^	1.73 × 10^−6^	3.02 × 10^−11^
F14	std	7.20 × 10^1^	2.23 × 10^4^	3.71 × 10^5^	7.65 × 10^4^	1.79 × 10^6^	8.00 × 10^4^	1.27 × 10^4^	3.25 × 10^5^
	avg	1.65 × 10^3^	1.18 × 10^4^	3.03 × 10^5^	6.83 × 10^4^	1.73 × 10^6^	7.24 × 10^4^	1.46 × 10^4^	3.10 × 10^5^
	median	1.64 × 10^3^	2.59 × 10^3^	1.03 × 10^5^	3.43 × 10^4^	1.17 × 10^6^	5.00 × 10^4^	1.12 × 10^4^	2.10 × 10^5^
	*p*-value	-	3.96 × 10^−8^	3.02 × 10^−11^	3.02 × 10^−11^	3.02 × 10^−11^	3.02 × 10^−11^	3.02 × 10^−11^	3.02 × 10^−11^
F15	std	1.12 × 10^3^	1.64 × 10^4^	1.16 × 10^7^	6.05 × 10^4^	4.20 × 10^4^	2.78 × 10^5^	1.29 × 10^4^	1.01 × 10^6^
	avg	2.61 × 10^3^	2.72 × 10^4^	3.59 × 10^6^	6.36 × 10^4^	7.45 × 10^4^	2.27 × 10^5^	1.52 × 10^4^	3.55 × 10^5^
	median	2.18 × 10^3^	2.48 × 10^4^	6.60 × 10^4^	5.35 × 10^4^	6.69 × 10^4^	1.13 × 10^5^	9.49 × 10^3^	8.16 × 10^4^
	*p*-value	-	3.02 × 10^−11^	3.02 × 10^−11^	7.39 × 10^−11^	3.02 × 10^−11^	3.02 × 10^−11^	9.26 × 10^−9^	3.02 × 10^−11^
F16	std	2.93 × 10^2^	5.58 × 10^2^	3.78 × 10^2^	3.76 × 10^2^	4.15 × 10^2^	2.21 × 10^2^	2.95 × 10^2^	3.72 × 10^2^
	avg	2.79 × 10^3^	3.08 × 10^3^	2.97 × 10^3^	3.03 × 10^3^	3.69 × 10^3^	3.71 × 10^3^	2.87 × 10^3^	3.86 × 10^3^
	median	2.85 × 10^3^	2.95 × 10^3^	2.91 × 10^3^	3.00 × 10^3^	3.60 × 10^3^	3.72 × 10^3^	2.91 × 10^3^	3.88 × 10^3^
	*p*-value	-	6.15 × 10^−2^	1.12 × 10^−1^	3.50 × 10^−3^	8.89 × 10^−10^	4.50 × 10^−11^	4.92 × 10^−1^	1.78 × 10^−10^
F17	std	1.94 × 10^2^	2.53 × 10^2^	2.43 × 10^2^	2.80 × 10^2^	2.84 × 10^2^	1.63 × 10^2^	2.66 × 10^2^	2.47 × 10^2^
	avg	2.11 × 10^3^	2.31 × 10^3^	2.18 × 10^3^	2.39 × 10^3^	2.67 × 10^3^	2.52 × 10^3^	2.33 × 10^3^	2.82 × 10^3^
	median	2.09 × 10^3^	2.28 × 10^3^	2.16 × 10^3^	2.37 × 10^3^	2.59 × 10^3^	2.54 × 10^3^	2.34 × 10^3^	2.84 × 10^3^
	*p*-value	-	7.70 × 10^−4^	1.58 × 10^−1^	1.75 × 10^−5^	7.38 × 10^−10^	3.20 × 10^−9^	4.71 × 10^−4^	1.96 × 10^−10^
F18	std	1.64 × 10^4^	1.02 × 10^6^	1.02 × 10^6^	7.46 × 10^5^	5.24 × 10^6^	2.00 × 10^6^	1.81 × 10^5^	2.24 × 10^6^
	avg	1.79 × 10^4^	3.33 × 10^5^	9.64 × 10^5^	5.52 × 10^5^	4.72 × 10^6^	2.74 × 10^6^	2.26 × 10^5^	1.41 × 10^6^
	median	9.19 × 10^3^	8.35 × 10^4^	6.42 × 10^5^	1.83 × 10^5^	2.90 × 10^6^	2.14 × 10^6^	1.69 × 10^5^	5.57 × 10^5^
	*p*-value	-	4.20 × 10^−10^	4.08 × 10^−11^	9.92 × 10^−11^	3.02 × 10^−11^	3.02 × 10^−11^	4.50 × 10^−11^	3.02 × 10^−11^
F19	std	9.86 × 10^2^	1.42 × 10^6^	6.35 × 10^7^	1.94 × 10^7^	4.17 × 10^6^	1.63 × 10^5^	1.88 × 10^4^	1.04 × 10^6^
	avg	2.51 × 10^3^	5.20 × 10^5^	1.68 × 10^7^	3.91 × 10^6^	4.00 × 10^6^	1.64 × 10^5^	1.74 × 10^4^	1.19 × 10^6^
	median	2.13 × 10^3^	7.38 × 10^4^	1.61 × 10^6^	8.50 × 10^4^	2.17 × 10^6^	1.16 × 10^5^	9.28 × 10^3^	9.15 × 10^5^
	*p*-value	-	3.69 × 10^−11^	3.02 × 10^−11^	3.02 × 10^−11^	3.02 × 10^−11^	3.02 × 10^−11^	3.81 × 10^−7^	3.02 × 10^−11^
F20	std	1.59 × 10^2^	1.58 × 10^2^	1.42 × 10^2^	1.86 × 10^2^	2.53 × 10^2^	1.31 × 10^2^	1.97 × 10^2^	1.54 × 10^2^
	avg	2.45 × 10^3^	2.49 × 10^3^	2.50 × 10^3^	2.61 × 10^3^	2.84 × 10^3^	2.97 × 10^3^	2.60 × 10^3^	3.00 × 10^3^
	median	2.42 × 10^3^	2.45 × 10^3^	2.52 × 10^3^	2.63 × 10^3^	2.87 × 10^3^	2.97 × 10^3^	2.61 × 10^3^	3.05 × 10^3^
	*p*-value	-	2.40 × 10^−1^	1.12 × 10^−1^	1.24 × 10^−3^	2.38 × 10^−7^	4.98 × 10^−11^	1.52 × 10^−3^	6.70 × 10^−11^
F21	std	4.40 × 10^1^	6.49 × 10^1^	3.05 × 10^1^	4.88 × 10^1^	5.58 × 10^1^	1.86 × 10^1^	5.25 × 10^1^	3.11 × 10^1^
	avg	2.51 × 10^3^	2.55 × 10^3^	2.45 × 10^3^	2.52 × 10^3^	2.57 × 10^3^	2.53 × 10^3^	2.51 × 10^3^	2.56 × 10^3^
	median	2.51 × 10^3^	2.54 × 10^3^	2.44 × 10^3^	2.52 × 10^3^	2.58 × 10^3^	2.53 × 10^3^	2.50 × 10^3^	2.56 × 10^3^
	*p*-value	-	3.39 × 10^−2^	6.05 × 10^−7^	3.40 × 10^−1^	4.64 × 10^−5^	1.22 × 10^−2^	8.77 × 10^−1^	7.20 × 10^−5^
F22	std	1.95 × 10^3^	1.68 × 10^3^	1.05 × 10^3^	1.83 × 10^3^	2.38 × 10^3^	2.95 × 10^3^	2.08 × 10^3^	8.03 × 10^2^
	avg	3.13 × 10^3^	6.25 × 10^3^	4.08 × 10^3^	3.64 × 10^3^	6.67 × 10^3^	5.26 × 10^3^	4.06 × 10^3^	3.66 × 10^3^
	median	2.30 × 10^3^	6.67 × 10^3^	3.66 × 10^3^	2.38 × 10^3^	7.64 × 10^3^	3.65 × 10^3^	2.31 × 10^3^	3.37 × 10^3^
	*p*-value	-	8.20 × 10^−7^	6.74 × 10^−6^	8.12 × 10^−4^	4.31 × 10^−8^	3.26 × 10^−7^	1.71 × 10^−1^	7.22 × 10^−6^
F23	std	7.44 × 10^1^	1.43 × 10^2^	5.67 × 10^1^	1.04 × 10^2^	8.56 × 10^1^	1.75 × 10^1^	6.52 × 10^1^	8.42 × 10^1^
	avg	2.91 × 10^3^	3.13 × 10^3^	2.88 × 10^3^	2.93 × 10^3^	3.08 × 10^3^	2.90 × 10^3^	2.87 × 10^3^	3.12 × 10^3^
	median	2.91 × 10^3^	3.09 × 10^3^	2.89 × 10^3^	2.92 × 10^3^	3.09 × 10^3^	2.89 × 10^3^	2.86 × 10^3^	3.12 × 10^3^
	*p*-value	-	2.61 × 10^−10^	1.37 × 10^−1^	5.49 × 10^−1^	7.77 × 10^−9^	1.15 × 10^−1^	2.07 × 10^−2^	1.78 × 10^−10^
F24	std	8.88 × 10^1^	1.26 × 10^2^	7.19 × 10^1^	1.03 × 10^2^	9.78 × 10^1^	2.04 × 10^1^	7.15 × 10^1^	8.17 × 10^1^
	avg	3.14 × 10^3^	3.24 × 10^3^	3.07 × 10^3^	3.10 × 10^3^	3.16 × 10^3^	3.07 × 10^3^	3.04 × 10^3^	3.23 × 10^3^
	median	3.13 × 10^3^	3.19 × 10^3^	3.04 × 10^3^	3.10 × 10^3^	3.15 × 10^3^	3.07 × 10^3^	3.03 × 10^3^	3.22 × 10^3^
	*p*-value	-	1.06 × 10^−3^	1.52 × 10^−3^	1.67 × 10^−1^	4.04 × 10^−1^	2.16 × 10^−3^	8.66 × 10^−5^	5.87 × 10^−4^
F25	std	2.46 × 10^1^	7.84 × 10^2^	1.41 × 10^2^	4.08 × 10^1^	3.17 × 10^1^	3.07 × 10^1^	1.61 × 10^1^	9.35 × 10^1^
	avg	2.91 × 10^3^	3.27 × 10^3^	3.20 × 10^3^	2.92 × 10^3^	2.97 × 10^3^	2.98 × 10^3^	2.90 × 10^3^	3.18 × 10^3^
	median	2.89 × 10^3^	2.98 × 10^3^	3.16 × 10^3^	2.92 × 10^3^	2.96 × 10^3^	2.98 × 10^3^	2.89 × 10^3^	3.15 × 10^3^
	*p*-value	-	1.43 × 10^−8^	3.02 × 10^−11^	1.41 × 10^−1^	1.31 × 10^−8^	2.61 × 10^−10^	4.21 × 10^−2^	3.02 × 10^−11^
F26	std	1.82 × 10^3^	1.77 × 10^3^	6.46 × 10^2^	1.02 × 10^3^	1.17 × 10^3^	6.40 × 10^2^	8.33 × 10^2^	1.36 × 10^3^
	avg	5.73 × 10^3^	7.38 × 10^3^	5.82 × 10^3^	6.16 × 10^3^	7.51 × 10^3^	6.03 × 10^3^	6.11 × 10^3^	7.61 × 10^3^
	median	6.37 × 10^3^	8.03 × 10^3^	5.71 × 10^3^	6.25 × 10^3^	7.79 × 10^3^	6.12 × 10^3^	6.23 × 10^3^	7.98 × 10^3^
	*p*-value	-	8.14 × 10^−5^	1.41 × 10^−1^	9.82 × 10^−1^	5.86 × 10^−6^	3.79 × 10^−1^	8.19 × 10^−1^	2.00 × 10^−5^
F27	std	4.32 × 10^1^	1.12 × 10^2^	6.16 × 10^1^	7.44 × 10^1^	1.09 × 10^2^	1.84 × 10^1^	2.68 × 10^1^	7.60 × 10^1^
	avg	3.31 × 10^3^	3.38 × 10^3^	3.34 × 10^3^	3.34 × 10^3^	3.37 × 10^3^	3.28 × 10^3^	3.25 × 10^3^	3.40 × 10^3^
	median	3.31 × 10^3^	3.37 × 10^3^	3.32 × 10^3^	3.32 × 10^3^	3.36 × 10^3^	3.28 × 10^3^	3.25 × 10^3^	3.39 × 10^3^
	*p*-value	-	1.38 × 10^−2^	1.54 × 10^−1^	3.33 × 10^−1^	3.03 × 10^−2^	2.84 × 10^−4^	1.36 × 10^−7^	2.15 × 10^−6^
F28	std	5.81 × 10^1^	8.79 × 10^2^	3.39 × 10^2^	5.20 × 10^1^	2.76 × 10^1^	6.04 × 10^1^	5.33 × 10^1^	3.10 × 10^2^
	avg	3.15 × 10^3^	3.60 × 10^3^	3.84 × 10^3^	3.29 × 10^3^	3.35 × 10^3^	3.38 × 10^3^	3.20 × 10^3^	3.83 × 10^3^
	median	3.10 × 10^3^	3.28 × 10^3^	3.74 × 10^3^	3.27 × 10^3^	3.34 × 10^3^	3.36 × 10^3^	3.20 × 10^3^	3.74 × 10^3^
	*p*-value	-	7.39 × 10^−11^	3.02 × 10^−11^	3.47 × 10^−10^	3.02 × 10^−11^	3.69 × 10^−11^	5.26 × 10^−4^	3.02 × 10^−11^
F29	std	2.73 × 10^2^	4.18 × 10^2^	3.23 × 10^2^	3.26 × 10^2^	3.55 × 10^2^	2.20 × 10^2^	3.12 × 10^2^	3.95 × 10^2^
	avg	4.04 × 10^3^	4.53 × 10^3^	4.22 × 10^3^	4.39 × 10^3^	4.93 × 10^3^	4.60 × 10^3^	4.07 × 10^3^	5.07 × 10^3^
	median	4.07 × 10^3^	4.44 × 10^3^	4.17 × 10^3^	4.33 × 10^3^	4.91 × 10^3^	4.61 × 10^3^	4.04 × 10^3^	5.02 × 10^3^
	*p*-value	-	8.88 × 10^−6^	4.36 × 10^−2^	7.66 × 10^−5^	2.61 × 10^−10^	5.00 × 10^−9^	7.62 × 10^−1^	1.21 × 10^−10^
F30	std	4.64 × 10^3^	5.84 × 10^7^	1.27 × 10^7^	3.19 × 10^6^	1.29 × 10^7^	9.47 × 10^5^	7.89 × 10^3^	1.43 × 10^7^
	avg	1.07 × 10^4^	1.98 × 10^7^	1.85 × 10^7^	2.85 × 10^6^	1.62 × 10^7^	1.24 × 10^6^	1.78 × 10^4^	1.72 × 10^7^
	median	8.64 × 10^3^	9.26 × 10^5^	1.47 × 10^7^	1.42 × 10^6^	1.19 × 10^7^	9.47 × 10^5^	1.76 × 10^4^	1.43 × 10^7^
	*p*-value	-	3.02 × 10^−11^	3.02 × 10^−11^	1.09 × 10^−10^	3.02 × 10^−11^	3.02 × 10^−11^	6.36 × 10^−5^	3.02 × 10^−11^
Friedman	Value	1.97 × 10^0^	5.38 × 10^0^	4.66 × 10^0^	4.00 × 10^0^	6.28 × 10^0^	4.72 × 10^0^	2.28 × 10^0^	6.72 × 10^0^
	Rank	1	6	4	3	7	5	2	8

**Table 3:** Performance Comparison of Baseline Models.

Model	MAE	MAPE	MSE	RMSE	R^2^	Runtime
SVM	0.5435	0.7561	0.4193	0.6476	0.9468	0.0056
BP	0.9616	1.3433	1.2230	1.1059	0.8449	3.9404
ELM	0.7474	1.0480	0.9478	0.9736	0.8798	0.0087
CNN	0.9825	1.3495	1.5477	1.2441	0.8037	8.6022
LSTM	0.6864	0.9552	0.6286	0.7929	0.9203	5.3814

**Table 4:** Parameter settings for the envelope structure of the baseline building model.

Type	Material Name	Thickness (mm)	Density (kg/m^3^)	Thermal Conductivity [W/(m·K)]	Specific Heat Capacity [kJ/(kg·K)]
Exterior Wall	Rammed Earth	400	2000	1.92	0.52
Roof	Tile Roof	30	1600	3.85	0.26
	Straw-Mud	40	1600	0.58	1.01
Window	Sheet Glass	3	2500	5.53	0.18
Floor	Rammed Earth	400	2000	1.66	0.6
Insulation Layer	EPS External Insulation	/	30	0.042	1.38

**Table 5:** Simulation Parameter Settings.

	Bedroom	Kitchen	Central Hall
Clothing Thermal Resistance/clo	1.7	1.7	1.7
Metabolic Rate/met	1	2	1.6
External Work/met	0	0	0
Relative Air Velocity, m/s	0.1	0.1	0.1

**Table 6:** Selection and Values of Energy Consumption Impact Factors.

	Window-Wall Ratio in East	Window-Wall Ratio in West	Window-Wall Ratio in South	Window-Wall Ratio in North	Sun Space Depth	Insulation Thickness in Outer Wall	Insulation Thickness in Roof Covering
Level 1	0.06	0.06	0.1	0.01	0.6	160	160
Level 2	0.1	0.1	0.15	0.05	0.9	170	170
Level 3	0.14	0.14	0.2	0.09	1.2	180	180
Level 4	0.18	0.18	0.25	0.13	1.5	190	190
Level 5	0.22	0.22	0.3	0.17	1.8	200	200
Level 6	0.26	0.26	0.35	0.21	2.1	210	210
Level 7	0.3	0.3	0.1	0.25	2.4	220	220

**Table 7:** Energy Consumption of 49 Combinations of Energy Consumption Influencing Factors.

Order	Window-Wall Ratio in East	Window-Wall Ratio in West	Window-Wall Ratio in South	Window-Wall Ratio in North	Sun Space Depth	Insulation Thickness in Outer Wall	Insulation Thickness in Roof Covering	Energy Consumption
1	0.06	0.06	0.1	0.01	0.6	160	160	7.91 × 10
2	0.06	0.1	0.15	0.05	0.9	170	170	7.54 × 10
3	0.06	0.14	0.2	0.09	1.2	180	180	7.28 × 10
4	0.06	0.18	0.25	0.13	1.5	190	190	7.09 × 10
5	0.06	0.22	0.3	0.17	1.8	200	200	6.94 × 10
6	0.06	0.26	0.35	0.21	2.1	210	210	6.81 × 10
7	0.06	0.3	0.4	0.25	2.4	220	220	6.71 × 10
8	0.1	0.06	0.15	0.09	1.5	200	210	7.31 × 10
9	0.1	0.1	0.2	0.13	1.8	210	220	7.14 × 10
10	0.1	0.14	0.25	0.17	2.1	220	160	7.00 × 10
11	0.1	0.18	0.3	0.21	2.4	160	170	7.25 × 10
12	0.1	0.22	0.35	0.25	0.6	170	180	7.13 × 10
13	0.1	0.26	0.4	0.01	0.9	180	190	6.73 × 10
14	0.1	0.3	0.1	0.05	1.2	190	200	7.46 × 10
15	0.14	0.06	0.2	0.17	2.4	170	190	7.45 × 10
16	0.14	0.1	0.25	0.21	0.6	180	200	7.32 × 10
17	0.14	0.14	0.3	0.25	0.9	190	210	7.03 × 10
18	0.14	0.18	0.35	0.01	1.2	200	220	6.70 × 10
19	0.14	0.22	0.4	0.05	1.5	210	160	6.55 × 10
20	0.14	0.26	0.1	0.09	1.8	220	170	7.33 × 10
21	0.14	0.3	0.15	0.13	2.1	160	180	7.59 × 10
22	0.18	0.06	0.25	0.25	1.2	210	170	7.02 × 10
23	0.18	0.1	0.3	0.01	1.5	220	180	6.74 × 10
24	0.18	0.14	0.35	0.05	1.8	160	190	6.99 × 10
25	0.18	0.18	0.4	0.09	2.1	170	200	6.86 × 10
26	0.18	0.22	0.1	0.13	2.4	180	210	7.62 × 10
27	0.18	0.26	0.15	0.17	0.6	190	220	7.49 × 10
28	0.18	0.3	0.2	0.21	0.9	200	160	7.19 × 10
29	0.22	0.06	0.3	0.05	2.1	180	220	7.02 × 10
30	0.22	0.1	0.35	0.09	2.4	190	160	6.87 × 10
31	0.22	0.14	0.4	0.13	0.6	200	170	6.78 × 10
32	0.22	0.18	0.1	0.17	0.9	210	180	7.41 × 10
33	0.22	0.22	0.15	0.21	1.2	220	190	7.19 × 10
34	0.22	0.26	0.2	0.25	1.5	160	200	7.40 × 10
35	0.22	0.3	0.25	0.01	1.8	170	210	7.14 × 10
36	0.26	0.06	0.35	0.13	0.9	220	200	6.70 × 10
37	0.26	0.1	0.4	0.17	1.2	160	210	6.87 × 10
38	0.26	0.14	0.1	0.21	1.5	170	220	7.62 × 10
39	0.26	0.18	0.15	0.25	1.8	180	160	7.44 × 10
40	0.26	0.22	0.2	0.01	2.1	190	170	7.18 × 10
41	0.26	0.26	0.25	0.05	2.4	200	180	7.03 × 10
42	0.26	0.3	0.3	0.09	0.6	210	190	6.94 × 10
43	0.3	0.06	0.4	0.21	1.8	190	180	6.74 × 10
44	0.3	0.1	0.1	0.25	2.1	200	190	7.50 × 10
45	0.3	0.14	0.15	0.01	2.4	210	200	7.25 × 10
46	0.3	0.18	0.2	0.05	0.6	220	210	7.14 × 10
47	0.3	0.22	0.25	0.09	0.9	160	220	7.25 × 10
48	0.3	0.26	0.3	0.13	1.2	170	160	7.02 × 10
49	0.3	0.3	0.35	0.17	1.5	180	170	6.85 × 10

**Table 8:** Evaluation Index Results Before and After Optimization.

Model	MAE (vs. SVR)	MAPE (vs. SVR)	MSE (vs. SVR)	RMSE (vs. SVR)	R^2^ (vs. SVR)
SVR	0.5435 (-)	0.7561 (-)	0.6476 (-)	0.4193 (-)	0.9468 (-)
IBKASVR	0.3285 (+39.6%)	0.4606 (+39.1%)	0.405 (+37.5%)	0.1641 (+60.9%)	0.9792 (+3.4%)
BKASVR	0.3689 (+32.1%)	0.516 (+31.8%)	0.4506 (+30.4%)	0.2031 (+51.6%)	0.9742 (+2.9%)
WOA-SVR	0.3666 (+32.6%)	0.5128 (+32.2%)	0.4478 (+30.9%)	0.2005 (+52.2%)	0.9746 (+2.9%)
DBO-SVR	0.3639 (+33.0%)	0.506 (+33.1%)	0.4416 (+31.8%)	0.195 (+53.5%)	0.9753 (+3.0%)
PSO-SVR	0.4572 (+15.9%)	0.6368 (+15.8%)	0.5489 (+15.2%)	0.3013 (+28.2%)	0.9618 (+1.6%)
GA-SVR	0.3582 (+34.1%)	0.5004 (+33.8%)	0.4151 (+35.9%)	0.1723 (+59.0%)	0.9781 (+3.3%)

## Data Availability

The original contributions presented in this study are included in the article. Further inquiries can be directed to the corresponding author.
